# Nonkin interactions between *Bacillus subtilis* soil isolates limit the spread of swarming deficient cheats

**DOI:** 10.1093/ismejo/wrae199

**Published:** 2024-10-07

**Authors:** Katarina Belcijan Pandur, Barbara Kraigher, Ana Tomac, Polonca Stefanic, Ines Mandic Mulec

**Affiliations:** Department of Microbiology, Biotechnical Faculty, University of Ljubljana, Ljubljana, Slovenia; Department of Microbiology, Biotechnical Faculty, University of Ljubljana, Ljubljana, Slovenia; Department of Microbiology, Biotechnical Faculty, University of Ljubljana, Ljubljana, Slovenia; Department of Microbiology, Biotechnical Faculty, University of Ljubljana, Ljubljana, Slovenia; Department of Microbiology, Biotechnical Faculty, University of Ljubljana, Ljubljana, Slovenia

**Keywords:** *Bacillus subtilis*, public goods, swarming, cooperation, cheats, kin discrimination, experimental evolution

## Abstract

Cooperative behaviours in human, animal, and even microbial societies are vulnerable to exploitation. Kin discrimination has been hypothesized to help stabilize cooperation. However, the mechanisms that sustain cooperative behaviour remain poorly understood. Here, we investigate the role of kin discrimination in limiting the spread of cheats in adjoining populations during surfactant-dependent cooperative swarming over surfaces using the bacterium *Bacillus subtilis* as a model organism. We show that mixing surfactant secreting cooperators and cheats that do not produce surfactants at 1:1 initial ratio quickly leads to cooperation collapse. However, when such common swarms encounter nonkin *B. subtilis* swarms, the proportion of the surfactant nonproducers decreases, suggesting that kinship dependent interactions may limit cheats’ advantage in an adjoining population. To further validate this finding, we subjected wild-type cooperators to multiple transient encounters with kin and nonkin swarms over 20 cycles of experimental evolution. The evolved populations exposed to nonkin swarms less frequently contained defective swarming phenotypes compared to those encountering kin swarms. Altogether, our results support the prediction that the spread of cheats in an adjoining bacterial population is impeded by kin discrimination interactions, which might have a role in stabilizing cooperative behaviour in evolving populations.

## Introduction

Bacteria have evolved intricate mechanisms to cooperate with each other, often forming complex communities and exhibiting collective behaviours. Although cooperation is widespread among organisms of different organizational levels [1], it is threatened by cheats (genetic mutants that gain a fitness advantage by exploiting public goods produced by cooperative individuals in the population; defined in Table 1) that benefit but do not contribute to the common good [2]. The theory predicts that the evolutionary persistence of cooperative behaviour must rely on mechanisms that limit the advantage of cheats and consequently stabilize cooperative acts [3–5]. For example, cooperation may be evolutionarily more stable if individuals preferentially cooperated with highly related (kin) individuals and avoid or fight less related (nonkin) conspecifics [5–7].

**Table 1:** Glossary with wording used in this paper.

**Cheats**	Genetic mutants that gain fitness advantage by exploiting public goods produced by cooperative individuals in the population. Surfactin is regarded as public good, therefore surfactin nonproducers that are not capable of swarming on their own but can swarm only by exploitation of surfactin produced by co-swarming surfactin producing strains are labelled as cheats in this paper.
**Exploitation**	Any degree of absolute gain to a non-cooperator from interacting with a cooperator, even if that gain does not lead to a relative fitness advantage of the non-cooperator.
**Evolved population**	A separate population of evolved clones that has been evolving during experimental evolution and has followed distinct evolutionary trajectory.
**Evolved clone**	A representative strain from a single colony isolated from evolved population after 20 reinoculation cycles of *in vitro* evolution.
**Contact strain**	Strains inoculated parallel to the focal strain or strain mixture on swarming agar, which exhibit different interactions due to kin discrimination. Isogenic contact strain (PS-216) and kin contact strain (PS-13) swarms merge with the focal strain’s swarm. However, nonkin contact strain (PS-218) forms a boundary at the contact with the swarm of the focal strain.

*B. subtilis*, a Gram-positive bacterium, exhibits a rich arsenal of social behaviours, including swarming, making it an excellent model for the study of cooperative group behaviour and its evolutionary stability [8]. Swarming is a collective movement performed by a variety of organisms from insects, fish, birds to microbes [9]. In some bacteria, swarming is a cooperative group motion by which cells migrate rapidly over surfaces, forming dynamic patterns of whirls and jets [10]. Cooperative movement is dependent on secreted surfactants, which are needed in addition to flagella to propel bacterial groups efficiently over surfaces [11]. The swarmer cells of *B. subtilis* secrete the lipopeptide antibiotic surfactin, a potent surfactant [12, 13], that reduces surface tension [11]. Surfactin production is governed by the *srfA* operon [14, 15], which is regulated by quorum sensing [16–18]. Surfactin producers release this public good into the surrounding environment, benefitting both the surfactin-producing cells and the nonproducing cells of the population [15, 19, 20]. However, the avoidance of metabolic investment by non-cooperative individuals may lead to their additional benefits, potentially destabilizing cooperation [1, 2]. Indeed, it was recently shown that Δ*srfA* mutants in a common swarm gain a reproductive fitness advantage by exploiting extracellular surfactin produced by the isogenic wild-type strain, thus behaving as cheats [19]. However, the stability of public goods exchange during bacterial cooperative behaviours in general and bacterial cooperative swarming in particular is still poorly understood. For example, surfactant sharing in the presence of cheats (Δ*srfA* mutants) during repeated growth cycles has not been yet experimentally investigated.

*B. subtilis* isolates are known to engage in kin discrimination-like behaviour [21]. We have previously shown swarms of closely related *B. subtilis* strains (kin, 99.93–99.99% average nucleotide identity (ANI)) merge upon encounter, whereas less related (nonkin, 98.73–98.84 ANI) strains form visible boundary lines between their swarms [21, 22], suggesting that *B. subtilis* swarmer cells preferentially cooperate with their clonemates and close kin, but avoid nonkin. Moreover, kin strains always formed a common swarm, whereas nonkin excluded each other by the positive frequency-dependent competitive exclusion [19]. *B. subtilis* isolates also engaged in kin discrimination-like behaviour in floating biofilms, with kin strains remaining closely intermixed, whereas nonkin segregated into well visible patches with one strain gaining dominance over the other [23]. These results support the prediction that kin discrimination mechanisms may be important for the evolutionary stability of cooperative behaviours. However, despite advances in our understanding of bacterial kin discrimination, several knowledge gaps persist in this area. One of these gaps concerns the role of kin discrimination in limiting the spread of cheats in naturally evolving swarms encountering individuals of different kinships. We predict that interactions at the nonkin boundary between the two swarms will decrease the advantage of swarming deficient mutant (Δ*srfA*) mixed with the parental strain. In addition, we predict that nonkin interactions at the contact area will limit the spread of swarming deficient mutants that evolve randomly during experimental evolution.

We analyzed the potential of cheats to collapse cooperative swarming and the effect of kin and nonkin interactions on the relative advantage of a clean Δ*srfA* mutant in the swarm mixed with the wild-type producer. Our results show that the advantage of surfactin cheats during co-swarming with isogenic producers rapidly leads to swarming collapse, resulting in a scenario akin to “the tragedy of the commons” [24, 25]. Furthermore, nonswarming surfactin cheats exhibited lower competitive index when common swarm composed of surfactin cheats and the isogenic surfactin producer was staged against the antagonistic nonkin strain than when it was staged against the isogenic or kin strains, suggesting that the interactions associated with kin discrimination hinder the advantage of Δ*srfA* mutant strain in the adjoining swarm. Finally, we address the effect of repeated contact with kin or nonkin swarms on the emergence of swarming deficient mutants during experimental evolution. The work provides insights into the role of kin discrimination interactions, which might be restricting the spread of swarming deficient mutants in the adjacent evolving populations during adaptation to the environment.

## Materials and methods

### Strains and media

The strains used in this study are described in Table 2. Strain BM1658 was prepared by transforming strain BM1336 [21] with pMS17 plasmid DNA (EM1096) [26] to obtain a strain with constitutively expressed yellow and red fluorescent proteins and two antibiotic resistances.

**Table 2:** Strains and plasmids used in this study.

Strain	Strain abbreviation	Genotype	Reference
BM1658	PS-216 Kn^R^ Sp^R^, focal strain	PS-216 *sacA*::p43-*mKate2* (Kn^R^) *amyE*::p43-*yfp* (Sp^R^)	This work
BM1629	PS-216 Kn^R^	PS-216 *sacA*::p43-*mKate2* (Kn^R^)	[26]
BM1345	PS-216 Sp^R^	PS-216 *amyE*::p43-c*fp* (Sp^R^)	[22]
BM1347	PS-218 Sp^R^	PS-218 *amyE*::p43-c*fp* (Sp^R^)	[22]
BM1350	PS-13 Sp^R^	PS-13 *amyE*::p43-c*fp* (Sp^R^)	[22]
BM1348	PS-196 Sp^R^	PS-196 *amyE*::p43-c*fp* (Sp^R^)	[22]
BM1328	PS-216 Cm^R^	PS-216 *sacA*::p43-*yfp* (Cm^R^)	[22]
BM1044	PS-216 Δ*srfA*	PS-216 *srfA*::*Tn917* (MLS^R^–resistance against Erythromycin 0.5 μg/ml and Lincomycin 12,5 μg/ml)	[18]
PS-216	PS-216	Wild type	[27]
PS-13	PS-13	Wild type	[27]
PS-218	PS-218	Wild type	[27]
BM1336		PS-216 *amyE*::p43-*yfp* (Sp^R^)	[21]
EM1096		*Escherichia coli* DH5 α (Amp^R^) with plasmid pMS17 (*sacA*::P43-*mKate2* (Kn^R^))	[26]

The strain abbreviations (Table 2) indicate the name of the parental wild-type strain and the specific antibiotic resistance and are referred to in the text as wild-type (w.t.) strains in the text. The mutant strain BM1044 [18], which does not produce surfactin, will be referred to below as PS-216 Δ*srfA*.

Bacteria were cultured in LB (Lennox) agar with 2% agar and LB (Lennox) liquid media supplemented with antibiotics (kanamycin 25 μg/ml–Kn, spectinomycin 100 μg/ml–Sp, chloramphenicol 10 μg/ml–Cm or MLS (erythromycin 0.5 μg/ml and lincomycin 12.5 μg/ml). Swarming agar, also known as B medium, was used for swarming experiments and was prepared with a final agar concentration of 0.7% [22].

M9 medium was used to grow cultures of isolated evolved clones (Table 1) for whole genome sequencing. M9 media was prepared by sterilization of 1 × M9 salts and addition of sterile solutions of MgSO_4_ × 7H_2_O (5 mM), CaCl_2_ (0.2 mM), glucose (1%), trace metal mix (0.25 × trace metal mix: 12.5 μM FeCl_3_, 5 μM CaCl_2_, 2.5 μM MnCl_2_ × 4H_2_O, 2.5 μM ZnSO_4_ × 7H_2_O, 0.5 μM CoCl_2_ × 6H_2_O, 0.5 μM CuCl_2_ × 2H_2_O, 0.5 μM NiCl_2_ × 6H_2_O, 0.5 μM Na_2_MnO_4_ × 2H_2_O, 0.5 μM Na_2_SeO_3_ × 5H_2_O, 0.5 μM H_3_BO_3_), BME vitamin mix (0.25×), sodium glutamate (0.04%), and casein hydrolysate (0.25%).

### Swarming assay

Each swarming plate was prepared by pouring exactly 15 ml of swarming agar onto a Petri dish and allowed to cool upright overnight. Overnight cultures were prepared from frozen cultures that were first plated, and single colonies were used to prepare overnight cultures. The overnight cultures were grown for 16 h at 37°C in LB or LB with an antibiotic. The plates were inoculated by spotting a 2 μl drop of the culture onto the swarming agar plate and allowed to dry. The plates were then incubated at 37°C and 80% relative humidity (RH) for 22–24 h until swarms formed.

### Spread of a clean surfactin deletion mutant in a common swarm

We tested the potential of PS-216 Δ*srfA* to cause the swarming collapse during cooperative swarming of surfactin producing strain and nonproducing strains. Overnight cultures of PS-216 and the nonswarming mutant strain PS-216 Δ*srfA* (MLS^R^) were mixed 1:1. We determined the relative frequency of the nonswarming mutant strain PS-216 Δ*srfA* (MLS^R^) that did not produce surfactin after co-swarming with the wild-type strain PS-216 on the swarming agar over three cycles of reinoculation by plating CFU on LB agar with selection for antibiotic resistance. The swarming assay was performed and after each reinoculation cycle, 20 agar cores were collected at the swarm edge using trimmed pipet tips. The agar cores with cells were resuspended in 250 μl saline solution (0.9% NaCl solution), vortexed vigorously and the resuspended cells (2 μl) reinoculated onto the centre of the fresh swarming agar. The number of cells in a sample was determined by CFU counts on LB agar or LB agar supplemented with MLS. Cycle of sampling and reinoculation were repeated until no swarming was observed on the plates after 24 h. To determine the temporal dynamics of the ratio between the nonswarming PS-216 Δ*srfA* (MLS^R^) mutant strain and the swarming wild-type strain PS-216 at the edge of a common swarm, the relative frequency of each strain was monitored in each cycle. The experiment was repeated in three independent experiments, with six populations per independent experiment.

To rule out the possibility that the collapse of swarming emerges spontaneously also in a population composed only of swarming proficient wild-type strain PS-216, we performed 20 cycles of sampling and reinoculation of the wild-type PS-216 populations on swarming agar. Although changes in swarming phenotype were observed, none out of the six evolving populations exhibited swarming collapse after 20 cycles of reinoculation (*n* = 6).

### Competitive index of the *srfA* mutant during co-swarming with its wild-type strain

We determined the competitive index of the mutant PS-216 Δ*srfA* in a common swarm with the wild-type strain PS-216 Kn^R^ after the contact with either isogenic (PS-216 Sp^R^), kin (PS-13 Sp^R^), or nonkin strain (PS-218 Sp^R^ and PS-196 Sp^R^). The competitive index (CI) is defined as the output ratio between the two strains divided by their input ratio [27, 28] and is used to determine the growth of the mutant strain compared to the wild-type strain during common colonization of prospective niches [29].

Overnight cultures of PS-216 Δ*srfA* (MLS^R^) and PS-216 (Kn^R^) were mixed in a 1:1 ratio and the relative frequency (abundance) of each strain was determined by plating on LB agar with antibiotics according to the resistance of each strain (MLS or Kn). The CFUs were counted after overnight incubation at 37°C. For the swarming assay, 2 μl of the mixed culture was inoculated at a distance of 3 cm with the isogenic strain PS-216 Sp^R^, the kin strain PS-13 Sp^R^, or the nonkin strain (PS-218 Sp^R^, or PS-196 Sp^R^). We sampled 20 agar cores using a trimmed 1 ml pipette tip at the point where the common swarm met the opposite swarm (PS-216 Sp^R^, PS-13 Sp^R^, PS-218 Sp^R^, or PS-196 Sp^R^). The samples were resuspended in 250 μl of saline solution (0.9% NaCl). Samples were vigorously mixed with a vortex mixer at highest speed (3000/min) at least twice for approximately 15 s, and the relative frequencies of co-swarming strains were again determined by plating on LB agar with selection for antibiotic resistance (MLS or Kn). We determined the competitive index (CI) of the focal strain PS-216 Δ*srfA* at the boundary with nonkin strains or at the merging point of kin strains. The competitive index (CI) was calculated by dividing *R_f_* by *R_i_* (Equation (1)). The initial ratio between the two co-swarming strains (PS-216 Δ*srfA* and PS-216 Kn^R^) (*R_i_*) was determined in the inoculum and the final ratio (*R_f_*) was determined at the swarm meeting area after the swarming cycle was completed (Equation (1)).


(1) \begin{equation*} CI=\frac{R_f}{R_i}=\frac{\frac{CFU_f\left(\mathrm{focal}\ \mathrm{strain}\right)}{CFU_f\ \left(\mathrm{co}-\mathrm{swarming}\ \mathrm{strain}\right)\ }}{\frac{CFU_i\left(\mathrm{focal}\ \mathrm{strain}\right)}{CFU_i\ \left(\mathrm{co}-\mathrm{swarming}\ \mathrm{strain}\right)}} \end{equation*}

Due to the high variability between CIs for the focal strains (PS-216 Δ*srfA* or PS-216 Cm^R^) in four independent experiments (Supplementary 1 Fig. S1), we determined the relative CI as the ratio between the CI of the focal strain when common swarm was staged against a kin or nonkin strain (CI_PS-218_, CI_PS-196_, CI_PS-13_) and its CI when common swarm was staged against the isogenic contact strain (CI_isogenic contact_) (Equation (2)). The ratios were consistent throughout independent experiments.


(2) \begin{equation*} {CI}_{relative}=\frac{{\mathrm{CI}}_{contact}}{{\mathrm{CI}}_{isogenic\ contact}} \end{equation*}

We performed four independent experiments, each with three replicates, and the relative competitive index was calculated for four independent experiments (*n* = 4). The log_10_ value of the relative CI would be zero (relative CI = 1) if competitive index was similar in the kin or nonkin interaction relative to the isogenic interaction. We calculated the 95% confidence interval of the mean log_10_ relative CI of four independent replicates and determined whether the value zero was included in the interval.

### Experimental evolution of strain PS-216 in contact with isogenic, kin, or nonkin strain

The experimental evolution of the focal strain PS-216 Sp^R^ Kn^R^ was performed in three steps. The swarming assay was performed by inoculating swarming agar (B medium) with the focal and contact strain (isogenic PS-216, kin PS-13, or nonkin strain PS-218) (Table 1) on opposite sides of the agar (at a distance of approximately 3 cm), allowing both strains to swarm over the agar overnight at 37°C and controlled humidity (80% RH). The next day, the bacterial cells were collected at the swarm encounter area by taking 20 agar cores with the trimmed pipette tips. The agar cores were resuspended in 0.5 ml of 0.9% saline solution (0.9% NaCl). The cells of the focal population were selected by growing (2% inoculum) in liquid LB media supplemented with antibiotics spectinomycin (Sp) and kanamycin (Kn) for 3 h. After selection, the swarming assay was repeated by reinoculation of the focal populations (2 μl) on swarming agar opposite to a contact strain. Populations sampled in the swarm encounter area were frozen in 7% glycerol at −80°C for later analyses. In total 20 cycles of re-inoculation and exposure to the contact strain (PS-216 (isogenic), PS-13 (kin), PS-218 (nonkin)) were performed in two separate experiments with at least six replicates per experiment for each contact strain (A-F and G-N).

### Screening for swarming defective phenotype in evolved populations

The evolved populations (Table 1) of the last (20th) evolutionary cycle (12 to 14 populations for each of the three contact strains PS-216, PS-13, and PS-218) were screened for the swarming defective phenotype. A total of 39 frozen populations (12 to 14 for each of three contact strains) were scraped into 100 μl of saline solution (0.9% NaCl), diluted up to 100-fold, plated onto the LB agar medium supplemented with kanamycin and spectinomycin (LB Sp Kn), and incubated overnight at 37°C for single colonies to grow. For each of the 39 evolved populations 40 to 96 colonies (Supplementary 2 Table S1) were randomly selected and resuspended in 50 μl of liquid LB Sp Kn medium. Two μl of this suspension was then inoculated onto the centre of the swarming agar plates and incubated for 24 h at 37°C and 80% RH to allow swarms to form. The evolved clones were defined as swarming if the swarming phenotype was the same or very similar to that of the parental strain, as impaired movement if the clone was capable of movement but formed a smaller swarming colony without an intricate dendritic pattern, and as nonswarming if a clone did not swarm on semisolid B medium and only formed a compact colony at the inoculation point.

The screening was also performed for selected populations of the 10th cycle. Evolved clones were selected from each evolved population after the 10th cycle of reinoculation and their swarming phenotype was determined after inoculation on swarming agar. We tested 40 to 96 evolved clones from each evolved population (Supplementary 2 Table S1).

Two-tailed Fischer’s exact test was performed to test whether kin or nonkin contact strain interactions are associated with the frequency of populations containing swarming deficient clones (IBM SPSS Statistics, Version 29.0, IBM Corporation 1989, 2015, 64-bit).

### Drop-collapse test

The drop-collapse test was performed according to previous protocols [30, 31]. In brief, frozen (−80°C) evolved clones and parental cultures were revitalized on LB agar and then grown overnight from single colonies in liquid LB by shaking at 200 rpm at 37°C for 16 h. The overnight culture (1 ml) was centrifuged, and the spent medium was transferred to fresh centrifuge tubes and diluted with MQ water in 96-well plate. Methylene blue (2 μl) was added to 80 ml of each dilution to increase the visual contrast of the droplets. 3–5 μl of mineral oil was pipetted onto the lid of a 96-well microtitre plate and incubated at room temperature for at least 1 h to allow the oil to spread in each well. Two μl of the spent media samples were pipetted onto the oil covered wells of the microtitre plate lid and after one minute of incubation, the droplets formed were imaged using a stereomicroscope (Leica WILD M10, Leica Mycrosystems, Inc., Danahe Corporation, Wetzlar, Germany). Droplet sizes were determined with ImageJ (ImageJ 1.53f51, Java 1.8.0_172 (64-bit), Wayne Rasband, National Institutes of Health, United States), using Vernier calliper as a scale for spatial calibration (determining the ratio of pixels to distance with the Set scale function in ImageJ). The concentration of surfactants in the spent media was determined using the standard curve obtained by measuring the droplet size of known surfactin sodium salt concentrations (Fujifilm Wako Pure Chemical Corporation, Osaka, Japan). Surfactin sodium salt was dissolved in PBS buffer and dilutions (80 μg/ml to 0.04 mg/ml) were prepared in MQ water. Two μl of methylene blue was added to increase the contrast of the droplets. Droplet size was determined using ImageJ as described above, and a standard curve was drawn (Microsoft Excel for Microsoft 365 MSO (Version 2204 Build 16.0.15128.20158) 64-bit), which was used to measure surfactant concentrations in the spent medium of the parental and evolved clones. The mean concentrations of surfactants produced by the evolved clones were compared to the mean surfactant concentrations of the parental strain using the independent sample *T*-test with *P* values adjusted using the Bonferroni correction method (Microsoft Excel for Microsoft 365 MSO (Version 2204 Build 16.0.15128.20158) 64-bit).

### Sequencing

Seven evolved clones originating from evolved populations in contact with either isogenic, kin, or nonkin were selected for sequencing. Twenty-one evolved clones were revitalized from frozen cultures on LB agar plates and grown overnight at 37°C. A single colony was inoculated into liquid LB medium and grown for 16 h at 37°C with shaking at 200 rpm. M9 medium was inoculated with an overnight culture (1% inoculum) and the culture was grown to an early exponential growth phase at 37°C with shaking at 200 rpm (approximately 4 h and OD_650 nm_ 0.2–0.3 a.u.). The resulting culture was pelleted by centrifugation (5 min, 10 000 g), the medium was removed, and the cells were washed with saline solution (0.9% NaCl). The cells were pelleted again by centrifugation (5 min, 10 000 g), the supernatant was removed, and the culture was frozen at −80°C. The frozen culture samples were sent for DNA sequencing (Macrogen Korea, Geumcheon-gu, Seoul, Korea).

Because DNA of sufficient quality and quantity for whole genome sequencing (WGS) could not be obtained from frozen culture samples of some evolved clones by Macrogene (Korea, Geumcheon-gu, Seoul, Korea), we isolated DNA from these strains (216-K54, 216-K106, 13-K57, 13-K82, and 13-K83) according to the following protocol. Single colonies of revitalized evolved clones were inoculated into liquid LB medium and incubated for 16 h with shaking at 200 rpm at 37°C. The culture was inoculated into fresh LB medium supplemented with 1% glucose and grown to early exponential phase (3.5 h at 37°C with shaking at 200 rpm). DNA was isolated from 2 ml of the bacterial culture using the EURx Basic DNA Purification Kit (EURx Sp. Z o.o., Gdansk, Poland) according to the standard protocol. The DNA was eluted twice with 50 μl of hot (80°C) elution buffer. DNA quality control was performed with the 4200 TapeStation System (Agilent, Santa Clara, CA, United States) according to the standard protocol using the Genomic DNA ScreenTape and TapeStation software (Agilent, Santa Clara, CA, United States) (kindly provided by prof. Dr P. Trontelj at the Department of Biology, Biotechnical Faculty, University of Ljubljana).

Sequencing of genomic DNA of 21 evolved clones and the parental strain was performed on the HiSeq X Ten System (Illumina) (Macrogen Korea, Geumcheon-gu, Seoul, Korea) using libraries prepared with the Illumina-Shotgun library (Illumina TruSeq DNA PCR-free [350 bp insert]), which generated 150 bp pair-end reads (genome sequences of the evolved clones are available in the NCBI database under BioProject accession number PRJNA1031373).

The genome of the parental strain PS-216, which was previously sequenced using an MiSeq System (Illumina) (genome sequence available in the NCBI database under BioSample accession number SAMN08637096) [22] and additionally sequenced by Pacific Biosciences RSII platform (PacBio) using a 10 kbp single-molecule real-time (SMRTbell) library (Macrogen Korea, Geumcheon-gu, Seoul, Korea).

### Sequence assembly and analysis

The genome of the parental strain PS-216 was assembled with short Illumina sequences and PacBio long-range sequences. Adapters of Illumina sequences, low-quality bases, and low-quality reads (Q < 25, shorter than 70 bp) were removed using Trimmomatic version v0.39 [32]. FastQC version v0.11.9 [33] was used to confirm the quality of the trimmed reads. No reads were flagged as poor quality, and no overrepresented sequences or Illumina adapters were present in Illumina sequences. Canu v2.2 [34] was used to remove low-quality bases in the reads of long-range PacBio sequences of the parental strain PS-216. The assembly of the parental strain PS-216 genome was performed de novo using Unicycler v0.4.9 [35] with default parameters and normal bridging mode. Quast 5.0.2 [36] was used to determine the quality of the assembly and the number of contigs. The assembly of the parental genome resulted in one contig (4 102 284 bp). Annotation was performed with PGAP 6.4 (NCBI Prokaryotic Genome Annotation Pipeline) [37].

The quality of the short reads of the evolved clones was first determined using FastQC version v0.11.9 [33] and Trimmomatic version v0.39 [32] was used to remove adapters, low-quality bases, and low-quality reads. Genome assembly of the evolved clones was performed *de novo* using Unicycler v0.4.9 [35] with default parameters and normal bridging mode.

Changes in the sequences of the evolved clones such as SNPs, insertions, deletions, and insertions or deletions of mobile genetic elements were identified using Breseq v036.1 [38], which maps the reads of the evolved clones onto the annotated reference genome (PS-216) using Bowtie 2 [39]. To distinguish between sequence gaps due to genome changes during experimental evolution and sequence gaps caused by incomplete assembly due to sequence repeats in the genome, we compared two types of mapping to the reference genome PS-216. First, we mapped the genomes of the evolved clones onto the genome of the parental strain PS-216 using the algorithm BWA-SW 0.7.17 [40], and then compared the data with the mapping of the short reads of evolved clones onto the reference genome obtained with Breseq. When short reads of the evolved genomes were mapped to sites where gaps were discovered with BWA-SW, we presumed that the observed gaps were a consequence of imperfect assembly and not true gaps in the genomes of the evolved clones. Sequence analysis with Trimmomatic, FastQC, Unicycler, and Quast was performed using the Galaxy web platform, specifically public server at galaxytrakr.org [41], version 21.09. The analysis with PGAP, Breseq (Bowtie2), and BWA-SW were performed in Linux Ubuntu 20.04.6 LTS (Canonical Ltd., United Kingdom).

## Results

### Collapse of swarming in kin populations occurs due to spread of surfactin cheats

**Figure 1:**
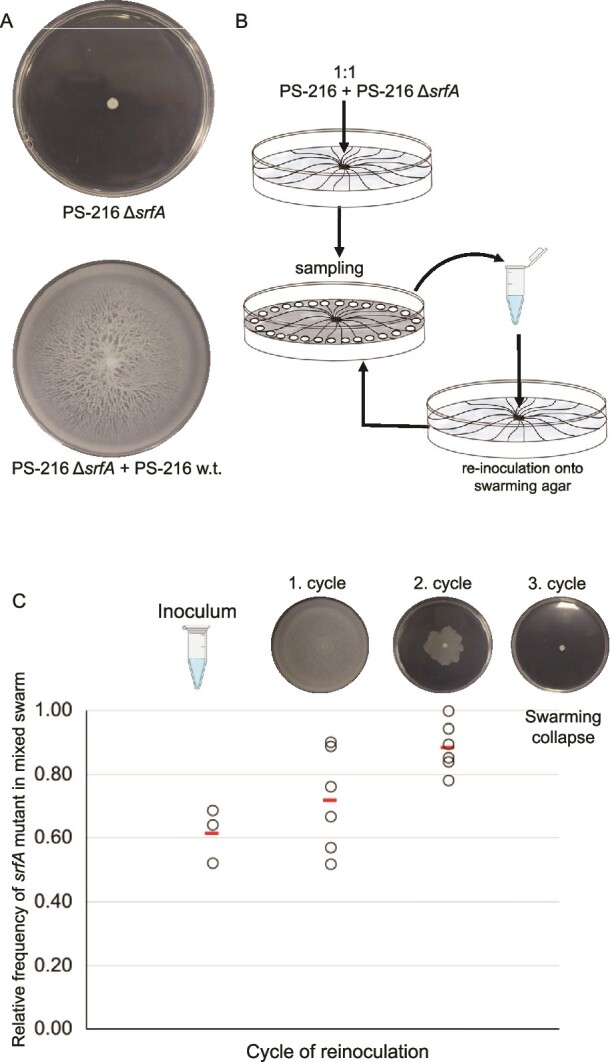
Dynamics of the relative frequencies of the surfactin mutant PS-216 during co-swarming with the wild-type strain PS-216 on swarming agar. (A) Surfactin nonproducing strain PS-216 Δ*srfA* on swarming agar, which cannot swarm, and nonproducing strain mixed with the wild-type strain, that form a common swarm and invade the entire surface of the swarming agar. (B) Schematic representation of the experiment. (C) The dot plot represents the relative frequency of the PS-216 Δ*srfA* strain in the sampled population at the swarm edge. The dots represent the relative frequency of PS-216 Δ*srfA* in the inoculum and in the sampled population at the edge of the swarm in the subsequent reinoculation cycles until the time that population could no longer swarm (swarming collapse). The lines represent the average relative frequency of PS-216 Δ*srfA* in all six populations. The independent experiment was performed with six replicate plates (*n* = 6). Results of all three independent experiments are presented in Supplementary 3 Fig. S2. Images above the relative frequencies for each reinoculation cycle exemplify the most common phenotype observed after each swarming cycle. (Fig. 1 was created in BioRender. Belcijan, K. (2024) BioRender.com/i71e741).

The collapse of cooperative behaviour can be attributed to the spread of cheats within a population. When a surfactant producing strain is mixed with its surfactin-deficient variant, the wild-type strain compensates for the inability of the mutant to swarm effectively (Fig. 1A) [19, 42]. Due to the fact that the mutant displays higher fitness during co-cultivation with its parental strain [19], we tested whether this exploitative dynamic would eventually undermine the cooperative swarming.

We evaluated the potential of PS-216 Δ*srfA* to cause the swarming collapse when co-swarming with the surfactin-producing strain by mixing the PS-216 *ΔsrfA* (MLS^R^) mutant and the surfactin-producing PS-216 wild-type strain in equal proportions (1:1 ratio) to give both strains equal possibility for gaining advantage during competition. This mixture was inoculated into the centre of the swarming agar. After 24 h, incubation samples were collected from the advancing swarm edge, and the relative frequency of each co-swarming strain was determined by selective plating. This sampling and reinoculation cycle were iterated until the swarming collapse occurred (Fig. 1B).

After the initial swarming cycle, the resultant common swarms were able to move over the entire surface of the swarming agar, in accordance with earlier findings [19, 43]. However, subtle blurring of dendritic structures was observed within these swarms. In the second cycle, the common swarm exhibited diminished size and lacked the well-defined dendrites characteristic of the PS-216 monoculture swarm. The relative frequencies of the surfactin mutant strain were mostly higher than 0.80. In the third cycle, a collapse of swarming occurred, with cells immobilized at the initial inoculation point (Fig. 1C and Supplementary 3 Fig. S2) and further sampling of the swarm edge was not possible.

### Local nonkin interactions hinder the advantage of the *srfA* mutant strain limiting its spread in cooperative groups

**Figure 2:**
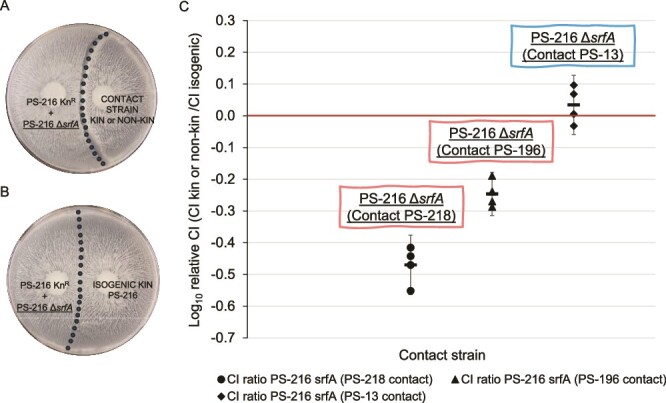
Log_10_ transformed relative competitive index of the surfactin mutant in the common swarm after contact with kin or nonkin swarm. (A and B) Schematic representation of the experiment where the surfactin nonproducing mutant (PS-216 Δ*srfA*) was mixed with the isogenic wild-type strain (PS-216 Kn^R^) in a 1:1 ratio and inoculated on one side of the agar (A) opposite to the kin contact strain (PS-13 Sp^R^), the nonkin contact strain (PS-218 Sp^R^, PS-196 Sp^R^), or (B) the isogenic contact strain (PS-216 Sp^R^). The strains were allowed to swarm for 24 h. We sampled at the point where the common swarm encountered the contact swarm and determined the CFU of each strain. We calculated the competitive index (CI) of the surfactin mutant strain PS-216 Δ*srfA*. (C) Log_10_ transformed relative CI represents the ratio between the CI of the focal strain PS-216 Δ*srfA* (underlined in schematic representation A and B) when common swarm was staged against a nonkin strain (CI_PS-218_ or CI_PS-196_) or a kin contact strain (CI_PS-13_) and its CI when common swarm was staged against an isogenic strain (CI_PS-216_) (Equation (2)). Log_10_ transformed relative CI was calculated for each of the four independent experiments, each performed in three replicates. The error bars represent the confidence interval (CI) of four replicates (*n* = 4).

According to the theory, kin discrimination can restrain exploitation and foster cooperation [6, 7, 44]. As established in previous studies on kin discrimination of *B. subtilis*, this mechanism can operate through territorial exclusion and antagonism between nonkin [19, 20, 44]. Therefore, it could also play a role in prevention of surfactin exploitation. To test the potential of nonkin strains to antagonize the nonproducing mutant and prevent the exploitation of the public good in the adjoining swarm, we evaluated the competitive index of PS-216 Δ*srfA* surfactin exploiters in a common swarm (PS-216 Δ*srfA* + PS-216 Kn^R^) upon encounter with a swarm of kin or nonkin cells, and compared it to the competitive index upon encounter with a swarm of isogenic cells. The inoculum was prepared by mixing the PS-216 Δ*srfA* mutant and the wild-type strain PS-216 in a 1:1 ratio. This mixture was inoculated on the swarming agar opposite to the inoculum of the contact strain (isogenic PS-216 Sp^R^, nonkin PS-196 Sp^R^, nonkin PS-218 Sp^R^, or kin PS-13 Sp^R^) (Fig. 2A and B). The sampling of the grown common swarm was performed at the swarm contact area and the CFU counts of the two co-swarming strains were determined. To assess the reproductive advantage of the mutant strain in common swarm relative to the wild-type strain, the competitive index (CI) of the PS-216 Δ*srfA* mutant was calculated by dividing the final ratio between the two co-swarming strains by their initial ratio (Equation (1), Supplementary 1 Fig. S1).

Competitive index (CI) of the nonswarming and surfactin nonproducing mutant PS-216 Δ*srfA* in a common swarm with the isogenic w.t. strain PS-216 Kn^R^ was higher in contact with the kin strain PS-216, than in contact with the nonkin swarm in all four separately performed experiments (PS-218 or PS-196) (Supplementary 1 Fig. S1B and C). Because of the variation observed in the calculated CI values across experiments, we calculated the relative CI values (calculated as CI in nonkin or kin interactions relative to CI in isogenic interactions) (Equation (2)), which were consistent across all independent experiments (Fig. 2C). The logarithmized relative CI value would be approximately zero (log_10_(relative CI) = 0) if the competitive index of the surfactin mutant strain in a common swarm was similar in kin or nonkin interactions relative to isogenic interactions.

Comparison of the relative competitive index (CI) of the *ΔsrfA* mutant in the common swarm when exposed to either kin or nonkin swarm unveiled a marked difference. The relative CI of the PS-216 Δ*srfA* mutant in the common swarm was less than zero when exposed to either of the two nonkin swarms (PS-218 Sp^R^ or PS196 Sp^R^) (95% confidence interval [−0.564; −0.376] and [−0.314; −0.178], respectively), suggesting that nonkin interactions reduce the advantage of the surfactin-deficient mutant in the common swarm (Fig. 2C). In contrast, the relative CI of the PS-216 Δ*srfA* mutant was close to zero (95% confidence interval [0.059; 0.128]) when exposed to the kin PS-13 swarm suggesting that kin and isogenic interactions have similar effects on the advantage of the surfactin-deficient mutant in the common swarm (Fig. 2C).

An additional control mixing two surfactin-producing strains PS-216 Cm^R^ and PS-216 Kn^R^ exposed to either isogenic (PS-216 Sp^R^) or nonkin strain (PS-218 Sp^R^) (Supplementary 4 Fig. S3B) indicated no effect of nonkin interactions on the wild-type strain as the relative CI was approximately zero (95% confidence interval [0.109; 0.211]) (Supplementary 1 Fig. S1A, Supplementary 4 Fig. S3C).

To further confirm that contact with the nonkin swarm was the cause for diminished CI of the nonswarming mutant PS-216 Δ*srfA*, we also compared its CI at the contact area and within the swarm area (approximately 1 cm from the swarm edge) (Supplementary 5 Fig. S4A). Relative CI based on CI of PS-216 Δ*srfA* at the contact area with the nonkin strain PS-218 (or isogenic strain PS-216) relative to the CI of PS-216 Δ*srfA* within the swarm area was calculated (Supplementary 5 Equation (S1)). We detected a decrease in the CI of PS-216 Δ*srfA* at the nonkin contact strain PS-218 relative to the CI of PS-216 Δ*srfA* within the swarm area (Supplementary 5 Fig. S4B). However, no difference was observed between the CI of PS-216 Δ*srfA* at the isogenic contact strain and within the swarm area (Supplementary 5 Fig. S4B).

### Antagonistic interactions may limit the spread of surfactin nonproducers during experimental evolution

**Figure 3:**
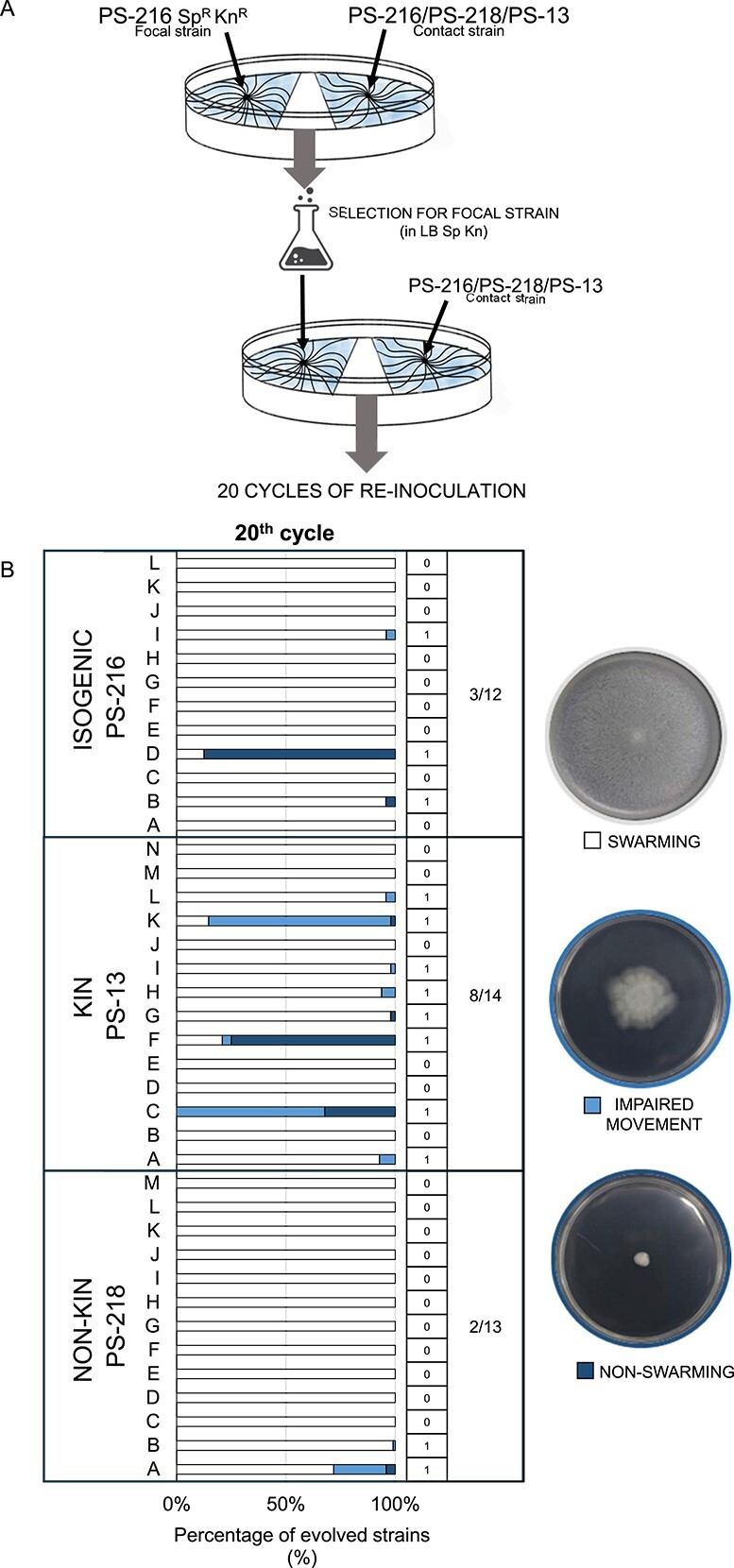
(A) Scheme of the experimental evolution of the *B. subtilis* strain PS-216 Kn^R^ Sp^R^, which was periodically exposed to a contact strain (isogenic PS-216, kin PS-13, nonkin PS-218) on swarming agar. At the contact area of two swarms, the evolved population was sampled and selected in LB with antibiotics and used for the next inoculation. (B) Swarming phenotypes of randomly selected evolved clones from 39 evolved populations periodically exposed to an isogenic, kin, or nonkin contact strain at the 20th cycle were determined. Evolved populations in which clones exhibited deficient swarming phenotype (nonswarming clones or clones exhibiting impaired swarming) are marked with “1” and evolved populations in which all tested clones exhibited swarming phenotype are marked with “0.” The overall number of populations containing swarming deficient clones according to the number of all screened evolved populations is summarized for each contact strain on the right. The images right of the graph are exemplifying the three swarming phenotypes. Fig. 3A was created in BioRender. Belcijan, K. (2024) BioRender.com/a33w561).

Previous results suggest that nonkin *B. subtilis* strains engage in antagonistic behaviours [19, 21–23]. Therefore, kin discrimination could have negative consequences for the spread of cheats, potentially shaping their evolution, and subsequentially aiding in stabilization of cooperative swarming behaviour in an adjoining population. To test this hypothesis, we conducted an experiment in which the focal PS-216 Kn^R^ Sp^R^ swarming population was periodically exposed to isogenic (PS-216), kin (PS-13), or nonkin (PS-218) swarms at the designated swarm encounter area (Fig. 3A). Based on the results of distinct relative competitive index obtained in kin or nonkin treatments, we predicted that the evolved mutants defective in surfactin production, and consequently impaired in swarming, will emerge more frequently in evolved populations with the history of kin encounters compared to evolved populations that were exposed to nonkin encounters.

Experimental evolution of PS-216 Kn^R^ Sp^R^ was performed on swarming B medium in six to eight replicate populations for each experimental variant in two separate experiments for 20 reinoculation cycles, equivalent to approximately 350 generations. The average number of generations during 24 h growth in liquid LB medium was 19.3 generations (95% confidence interval [23.4; 15.2]), whereas growth on B medium yielded an average of 17.5 generations (95% confidence interval [19.8; 15.3]) (Supplementary 6 Fig. S5). We also determined the mutation rate (see Supplementary 7 for a detailed description) for wild-type strain PS-216 being 3.72 × 10^−9^ mutations per cell division in LB media and 4.28 × 10^−9^ mutations per cell division during growth on swarming agar (Supplementary 7 Fig. S6).

Evolved clones were isolated form the 20th reinoculation cycle of each evolved population and tested for their swarming proficiency (swarming, impaired swarming, nonswarming). Overall, nonswarming clones and clones exhibiting impaired swarming (clones with deficient swarming) emerged in eight out fourteen (57%) populations in kin (PS-13) treatment and in two out of thirteen (15%) populations exposed to nonkin (PS-218) treatment (Fig. 3B, dark blue and light blue). There was a significant association between emergence of clones with deficient swarming phenotype and kin or nonkin treatment (Fischer’s exact test, *P* value = 0.046). However, we find that in isogenic (PS-216) treatment three out of twelve (25%) populations evolved cheats, which is not significantly different from the two out of thirteen in nonkin (PS-218) treatment. Therefore, additional factors besides kinship-driven interactions may have a role.

We additionally examined the frequency of clones that exhibited impaired swarming or nonswarming evolved clones (clones with deficient swarming) in four evolving populations after the 10th cycle of experimental evolution, including populations that exhibited a change in swarming phenotype at the 20th cycle (Supplementary 8 Fig. S7). Clones with deficient swarming phenotype were detected in two out of four screened populations in contact with the kin strain PS-13, two out of four screened populations in contact with the isogenic strain PS-216, and in one out of four screened populations in contact with the nonkin strain PS-218 (Supplementary 8 Fig. S7). The results are in line with conclusions from the results of 20th cycle.

The relative frequency of evolved clones with swarming deficient phenotype might depend on their overall fitness in the evolving population. Therefore, we tested the fitness of selected evolved clones in common swarms with the parental strain PS-216. The competitive index of the nonswarming evolved clones was variable and not consistent with the spread of the nonswarming clones in the corresponding evolved populations. For example, clones from population PS-13 C, which had the highest CI, did not spread as well as clones from population PS-13 F or population PS-216 D (Supplementary 9 Fig. S8 and Fig. 3B). To further delineate genetic bases of the mutants, we performed the whole genome sequencing and subsequent analyses.

### Mutations in the *srfA* operon in the evolved populations

Defects in swarming can be the consequence of different deleterious mutations, including mutations affecting flagellar function, chemotaxis, or surfactin production [45]. Of these, only surfactin production is considered *bona fide* social trait, whereas the other two are private traits associated with an individual cell [46, 47]. For each of the three types of evolved populations (isogenic, kin, or nonkin), we selected seven clones for sequencing. To determine the genetic causes of the nonswarming phenotype, we performed whole genome sequencing of 21 selected evolved clones. These clones exhibited either a change in swarming behaviour or retained the parental swarming pattern.

We found that two evolved clones (216-K54 and 216-K105) that had lost the ability to swarm and were in contact with the isogenic strain PS-216 had a deletion of 13 base pairs (bp) in *srfAB* (encoding surfactin synthase subunit 2 [48]). Furthermore, a mutation at position 2280 causing a stop codon in *srfAA* was identified in clone 216-K6. Although clone 216-K6 was capable of swarming, it did not form dendrites, as is typically associated with the wild-type PS-216 swarm. Of the clones isolated from populations interacting with the related strain PS-13, five carried a mutation in *srfAB* (13-K15, 13-K27, 13-K31, 13-K57, and 13-K41)*.* The nonswarming clones EV13-K41 and EV13-57 carried a single nucleotide polymorphism at position 1900 that caused a stop codon in *srfAB,* and clones 13-K15, 13-K27, and 13-K31 had a missense substitution (Leu496Pro) in *srfAB*. Finally, the evolved clone 218-K2, which was the only one isolated from the population in contact with the nonkin strain (PS-218) carried a major deletion of over 10 kbp in *srfAB* (including *comS*) (Table 3).

**Table 3:** Table of all mutations detected in the evolved clones. The evolved clones' contact strain is indicated on the left and their swarming phenotype marked by the clones’ name (^a^swarming, ^b^impaired swarming, and ^c^nonswarming).

Contact strain	Strain	Evolved population	Mutation in srf operon	Other common mutations						Unique mutations
PS-216	**216-K1^a^**	A		*oppA* (Y142*)						*bmr3* (M351K), *yknY* (Δ 1 bp), *spoIIM* (Δ 1 bp)
	**216-K6^a^**	B	*srfAA* (W2280*)							*ywkF* (G41A), *ywzG* (Δ 7 bp), *yxnA* (G214A), *tcyB* (I40T), *queA* (M130I), Δ9 622 bp (*sipT, ykoA, ykpA, ykpB, ampS, ykpC, mreBH, abh, kinC*)
	**216-K54^c^**	D	*srfAB* (Δ 13 bp)	*mnmE* (G327R)	*yhfT* (I380M)	*pksL* (A996E)	*gltD* (G194S)	*yqjN* (V265I)	*levB* (A282S)	
	**216-K15^a^**	E		*ahpC* (W170R)	*resC* (A64V)					*yxcE* (H135R)
	**216-K105^c^**	D	*srfAB* (Δ 13 bp)	*mnmE* (G327R)	*yhfT* (I380M)	*pksL* (A996E)	*gltD* (G194S)	*yqjN* (V265I)	*levB* (A282S)	
	**216-K106^a^**	F								*glpK* (A308V), *ywnA* (C- > T, −34)
	**216-K12^a^**	E								*citH* (G300D), *dctP* (A367V), *slp* (Δ 1 bp), *yrkQ* (F348Y), *licR* (N203T), *yxkI* (V147F)
PS-13	**13-K15^c^**	C	*srfAB*(L496P)	*oppA* (Δ 1 bp)	*catD* (A110V)	*katA-G* (−68)	*appA* (Q218*)			*csbC* (M1V)
	**13-K27^b^**	C	*srfAB*(L496P)	*oppA* (Δ 1 bp)	*catD* (A110V)	*katA-G* (−68)	*appA* (Q218*)			*rpoC* (T1143K)
	**13-K31^c^**	C	*srfAB*(L496P)	*oppA* (Δ 1 bp)	*catD* (A110V)	*katA-G* (−68)	*appA* (Q218*)			
	**13-K57^c^**	F	*srfAB*(Q1900*)	*ahpF* (G495D)	*pckA* (R20L)	*bdhJ* (S333Y)				
	**13-K41^c^**	F	*srfAB*(Q1900*)	*ahpF* (G495D)	*pckA* (R20L)	*bdhJ* (S333Y)				*papB*(Y155*)
	**13-K82^a^**	E								*degU* (G224R), *oppA* (Δ 1 bp), *kinC* (K72E), *prkC* (A445E), *bcbE* (M278I), *ytoQ* (D79N), tlpB (S320G)
	**13-K83^a^**	D								
PS-218	**218-K2^b^**	A	Δ*srfAB* (~Δ10kbp)							*efeM* (E340K), *perR* (P22S), *rapA* (D194G), *xkdV* (S330L)
	**218-K16^a^**	D		skin (Δ 48033 bp)	*yisY* (E254G)	*yjcK* (A156P)	*ylqG* (P457Q)	*epsE* (Δ 1 bp)	*yrdP* (Δ 1 bp)	
	**218-K20^a^**	F		skin (Δ 48033 bp)	*yisY* (E254G)	*yjcK* (A156P)	*ylqG* (P457Q)	*epsE* (Δ 1 bp)	*yrdP* (Δ 1 bp)	
	**218-K27^a^**	F		skin (Δ 48033 bp)	*yisY* (E254G)	*yjcK* (A156P)	*ylqG* (P457Q)	*epsE* (Δ 1 bp)	*yrdP* (Δ 1 bp)	
	**218-K12^a^**	D		*ahpC* (W170R)	*resC* (A64V)					hypot. Prot. (H135Y)
	**218-K56^a^**	F		skin (Δ 48033 bp)			*ylqG* (P457Q)		*yrdP* (Δ 1 bp)	*rhgX* (ins. A), *yfkN* (A256G)
	**218-K57^a^**	E								*glnQ* (E25Q), *essC* (E1425K), *fabZ* (D126Y), *cidR* (R4H), *yybG* (P47S)

The nonswarming clones within the same evolved population carried the same mutation, but clones from different populations carried distinct mutations (Table 3). This indicates that they evolved independently within each population, which is consistent with the observation that the evolved clones also have variable CI during co-swarming with the parental strain (Supplementary 9 Fig. S8 and Fig. 3B).

In total, we have found five different mutations in the *srfA* operon in all sequenced genomes, one of which originated from the population with the nonkin encounters. Although many genes contribute to swarming [45], none of the mutations outside the *srfA* operon (such as the mutation in *epsE*, which is involved in the production of the major biofilm matrix polysaccharide, or in the response regulator *degU*) resulted in a nonswarming or impaired swarming phenotype (Table 3) [49, 50]. However, other mutations present in the genomes of the evolved clones can affect the advantage of evolved clones in the evolving populations and therefore their ability to spread in the evolving population (Table 3 and Supplementary 9 Fig. S8).

### Evolved clones with defective swarm phenotype show impairment in surfactant production

Sequencing of the evolved clones revealed mutations in the *srfA* operon, which implied that the deficient swarming phenotype is caused by reduced surfactin production. To further verify this prediction, we randomly selected 20–25 clones from each of the three variants of experimental evolution (20th reinoculation cycle) and tested them for surfactin production by a drop collapse test according to the surfactin standard [30, 31].

**Figure 4:**
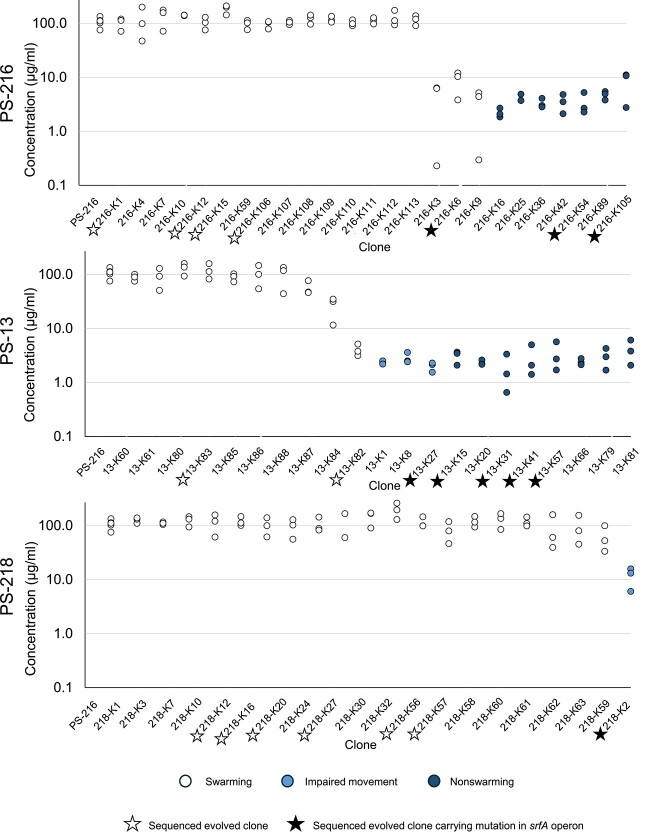
Surfactant concentrations produced by selected evolved clones in contact with the isogenic strain (PS-216), the kin strain (PS-13), and the nonkin strain (PS-218). The colour of dots indicates the swarming phenotype of the evolved clones. Sequenced clones are marked with an empty star next to the clones’ name and the star is full if an evolved clone carried a mutation in the *srfA* operon. The surfactant concentrations in the spent media of the evolved clones were measured in three independent experiments (*n* = 3). The surfactant concentrations produced by parental strain PS-216 were measured in twelve independent experiments (*n* = 12).

All tested nonswarming clones produced significantly less surfactants than the parental strain PS-216 (independent sample *T*-test, *P* values adjusted with Bonferroni correction, Fig. 4 and Supplementary 10 Table S2). Most of the evolved clones with no change in swarming proficiency produced similar amounts of surfactants as the parental strain PS-216 (Fig. 4 and Supplementary 11 Table S3). But there were a few exceptions, such as 216-K3, 216-K6, 216-K9, which evolved in contact with the isogenic strain PS-216, and 13-K82, 13-K84, 13-K87, which evolved in contact with kin strain PS-13, which produced less surfactants but were still capable of swarming (Fig. 4). Among these six indicated clones, the three clones (216-K3, 216-K6, 216-K9) colonized the swarming agar completely but did not form intricate dendrites pattern as the parental strain PS-216. One of the three evolved clones (216-K6) was also sequenced and was found to carry the mutation in *srfAA*, which presumably reduced the levels of surfactin production, but still allowed swarming (Fig. 4 and Supplementary 11 Table S3).

Altogether, only one out of twenty tested clones from the evolved population in contact with the nonkin contact strain was found to be swarming deficient, whereas a higher proportion of swarming deficient clones were found within the isogenic and the kin contact strain population (seven out of twenty five and eleven out of twenty one, respectively), which is consistent with the previous results indicating the role of nonkin interactions in limiting the spread of swarming deficient clones.

## Discussion

Cheats exploit the work of others by consuming shareable resources that are costly to produce but benefit the entire population [2]. It is intriguing to understand what makes bacterial cooperation persist despite the presence of cheats. Here we experimentally demonstrate that kinship-dependent interactions have an important role in maintaining cooperative swarming in adjoining swarming populations. We show that when surrounded by kin cooperators, the cheats that do not contribute surfactin (clean surfactin deletion mutant) quickly spread and cause the collapse of cooperative swarming. However, the outcome changes if the cheats in the population are exposed to nonkin swarm, which significantly diminishes surfactin mutants’ competitive index. Finally, we test the potential of this finding to play a role in protection from the cheats' invasion by experimental evolution. Results indicate that repeated transient contact with nonkin swarm may affect the spread of swarming deficient surfactin exploiters that evolved in focal swarms during adaptation to the environment.

The swarming motility of *B. subtilis* strains is supported by the surfactin production, which acts as a public good [15, 42, 47]. Even though public goods are shared and freely available, they can be easily exploited by cheats that benefit from public goods but do not invest in their production [2]. Therefore, nonproducers can outgrow producers in co-culture and cause the “tragedy of the commons” [24, 25]. Previous work has revealed that surfactin nonproducing strain can exploit surfactin produced by the wild-type strain during the first swarm cycle [19, 43]. These surfactin nonproducers also exhibit fitness advantage during co-swarming with the wild-type strain on swarming agar [19, 43] and during sliding motility without flagella [47]. Our results align with these observations, but additionally show that surfactin cheats, when mixed with the isogenic *B. subtilis* surfactin producers in a 1:1 ratio, quickly outgrow the producers and cause the collapse of the collective swarming already at the third re-inoculation cycle. In contrast, the *Pseudomonas aeruginosa* swarming system restricts the spread of surfactant cheats by metabolic prudence. Specifically, the rhamnolipid production is limited only to the stationary phase, where gain of fitness advantage of rhamnolipid cheats is not possible [51]. Moreover, *B. subtilis* NCBI 3610, which produces less surfactin than PS-216, is also better equipped to resist the spread of surfactin exploiters [43].

In contrast to the cheating-induced cooperation collapse of *B. subtilis* swarming, transient nonkin interactions decreased the fitness advantage of the clean surfactin deletion mutant co-swarming with the wild-type surfactin producer in a 1:1 initial mixture. In addition, we followed the emergence of surfactin mutants in evolving wild-type populations in contact with isogenic, kin, or nonkin swarms. The emergence of cheats during experimental evolution is the expected evolutionary outcome of adaptation to the environment, as previously shown for *Myxoccus xanthus* [52, 53]. However, whether repeated contact with isogenic, kin or nonkin contact swarms differentially affects the spread of nonswarming evolved clones that arise during evolution in adjoining swarm has not been tested previously. The results show statistically significant difference between nonkin and kin treatments, indicating that surfactin mutants emerge more frequently in populations when exposed to kin interactions as compared to nonkin. However, the difference between isogenic and nonkin treatments was not statistically significant. Although kinship driven interactions seem to contribute to frequency of swarming deficient clones in focal swarms during experimental evolution, we cannot exclude other factors, such as strain specific traits, to contribute to spread of swarming deficient clones. It is possible that under specific experimental conditions that involve swarm interactions at the meeting point of two swarms, where nutrients are limited, and cell density is high, swarming cells may potentially be capable of differentiating between self and kin and trigger additional policing mechanisms in the isogenic treatment. Future experiments are needed to test this speculation.

One might argue that the nonswarming clones within the kin treatment were fitter than those in the nonkin treatment and consequently spread to higher frequency. However, this reasoning is unlikely, as the competitive index of swarming deficient clones, when co-swarming with a swarming proficient wild-type strain, does not correlate well with their prevalence in the evolved populations from which they were isolated (Supplementary 9 Fig. S8). Furthermore, results of experimental evolution are supported by the previous results showing that nonkin contact affects the advantage of the clean surfactin cheat co-swarming with the wild-type surfactin producer (Fig. 2).

Sequencing of the evolved nonswarming clones confirmed our prediction that swarming deficiency is associated with reduced surfactin production as it uncovered five distinct mutations in the *srfA* operon, specifically in the *srfAA* and *srfAB* genes. Because surfactin is required for the colonization of synthetic B medium (swarming agar) [14, 42, 54], the swarming motility of such strains was impaired or even absent. Additionally, we detected significantly lower concentrations of surfactants in the spent media of the nonswarming evolved clones, suggesting that the detected mutations in the *srfA* operon are responsible for the nonswarming phenotype. Because the nonswarming evolved clones cannot swarm without exploiting the surfactin producers, they can be considered either as cheats or exploiters.

Evolving populations continued to swarm efficiently even after the 20th cycle, despite the spread of surfactin exploiters, likely because the cheats had not yet accumulated to a sufficient proportion for the associated costs to the cooperative strain to become high enough to cause the cooperation collapse. Sequencing data reveal that clones sampled from the same evolved population (20th cycle), while carrying the same mutation in *srfA* operon, also exhibit diverse mutations in other genes. Thus, genetically distinct evolved clones might hinder the cooperative trait mildly leading to a slower spread compared to the clean surfactin mutant.

The mechanism by which kin discrimination might limit the spread of swarming deficient mutants remains unclear. The lower competitive index of clean surfactin mutants after a nonkin encounter and their failure to spread during experimental evolution could be caused by different molecular mechanisms.

One of the explanations for the limited spread of surfactin mutants could be an increased sensitivity of surfactin mutants to nonkin attack. Previous research suggests that surfactin producing *B. subtilis* strains accumulate the stress phospholipid cardiolipin [55–57], which contributes to surfactin tolerance [55]. The mutant of *B. subtilis* lacking cardiolipin in the cytoplasmic membrane was more susceptible to surfactin than the wild type [55]. Furthermore, the surfactin nonproducing strain 168 stopped growing when inoculated on surfactin containing agar, but grew on agar without surfactin [56]. However, the presence of surfactin in the growth medium of surfactin nonproducing strain—168 also leads to the changes in the ratio between other phospholipid classes in the membrane, which also increase resistance to surfactin induced permeabilization [56, 57]. Similarly, a higher sensitivity to stress (osmotic, thermal, and heavy metal stress) was observed in *P. aeruginosa* lacking exoproteases. These quorum sensing deficient cheats were less resistant to oxidative stress, which caused decrease in the proportion of cheats in the population that was otherwise vulnerable to their invasion [58].

We have recently shown that kin discrimination promotes horizontal gene transfer (HGT) in *B. subtilis* by upregulating competence genes important for extracellular DNA uptake and transformation [22]. In addition, it was recently proposed [59] that horizontal gene transfer (HGT) is an important mechanism for enforcing cooperation in bacterial populations [60]. As a swarming deficient mutant can arise in a cooperating population through a random loss-of-function, HGT can convert the surfactin deficient clone into a cooperator by reintroducing a lost cooperative gene and rescuing cooperation [60]. Furthermore, HGT has previously been shown to function as a DNA repair mechanism [59, 61]. Therefore, increased HGT between nonkin strains at the boundary between swarms relative to decreased HGT between kin swarms could contribute to the decreased number of surfactin mutants at the boundary. Additionally, swarming deficient clone that evolved in the nonkin treatment and carries a deletion in the *srfA* operon also lacks the competence gene *comS*, suggesting that clone also lost the ability to take in DNA and repair the mutation.

Our research shows that random mutations in the *srfA* operon occur during the evolution of *B. subtilis* on the swarming agar. Such mutants could exhibit higher reproductive advantage than the wild type and therefore swiftly spread in the population of surfactin producers. However, not all evolved mutants were found to be true cheats with a fitness advantage, some were only exploiters that benefited from co-swarming with the surfactin producer but did not have a fitness advantage over the producer. In addition, we observed an indication that transient nonkin contacts at the swarm encounter area hinder the advantage of surfactin nonproducing mutants and restrict their spread, which suggests that nonkin swarm contact may benefit the rival nonkin population. Therefore, the described “reciprocity” between antagonistic populations might be involved in stabilizing cooperation in microbial populations in the natural environment. Most importantly, we show that nonkin interactions modify the evolutionary outcome of cooperative behaviour by the potential of nonkin interactions to hinder the spread of randomly emerging surfactin deficient mutants during experimental evolution on swarming media.

## Supplementary Material

SUPPLEMENT_ISMEJ_(revision3)_2_wrae199_(KBP_corrections)

## Data Availability

All data that support the findings of this study are available on Figshare platform (available under DOI: 10.6084/m9.figshare.27144288 (Fig. 1), 10.6084/m9.figshare.27144273 (Fig. 2), 10.6084/m9.figshare.27144282 (Fig. 3) and 10.6084/m9.figshare.27144276 (Fig. 4). Genome sequences are available in the NCBI database under BioProject accession number PRJNA1031373 and BioSample accession number SAMN08637096.
