# Supplementary material for: Nonkin interactions between Bacillus subtilis soil isolates limit the spread of swarming deficient cheats
Source: ISME J. 2024 Oct 7;18(1):wrae199. doi: 10.1093/ismejo/wrae199 (PMC11523185; doi:10.1093/ismejo/wrae199)
Supplement: SUPPLEMENT_ISMEJ_(revision3)_2_wrae199_(KBP_corrections) [file supplement_ismej_(revision3)_2_wrae199_(kbp_corrections).docx]

**Supplemental material**

**Nonkin interactions between *Bacillus subtilis* soil isolates limit the spread of swarming deficient** **cheats**

Katarina Belcijan Pandur^1^, Barbara Kraigher^1^, Ana Tomac^1^, Polonca Stefanic^1^*, Ines Mandic Mulec^1^*

^1^Department of Microbiology, Biotechnical Faculty, University of Ljubljana, Ljubljana, Slovenia

* Corresponding authors: Ines Mandic Mulec, [ines.mandicmulec@bf.uni-lj.si](mailto:ines.mandicmulec@bf.uni-lj.si) and Polonca Stefanic, [polonca.stefanic@bf.uni-lj.si](mailto:polonca.stefanic@bf.uni-lj.si), Department of Microbiology, Biotechnical Faculty, University of Ljubljana, Večna pot 111, Ljubljana, 1000, Slovenia

**Supplementary 1: Competitive index of PS-216 Δ*srfA* mutant depending on kin or nonkin contact strain**

We determined the competitive index of the focal mutant PS-216 Δ*srfA* in a co-swarm with the wild‑type strain PS-216 Kn^R^ after the contact with the isogenic (PS-216 Sp^R^) or nonkin strain (PS-218 Sp^R^).

Overnight cultures of PS-216 Δ*srfA* (MLS^R^) and PS-216 (Kn^R^) were mixed in a 1:1 ratio, and the frequency of each strain was determined by plating on LB agar with antibiotics according to the resistance of each strain (MLS or Kn, respectively). The CFUs were counted after overnight incubation at 37°C. During swarming assay 2 µl of the mixed culture was inoculated opposite the kin strain PS-216 Sp^R^ or the nonkin strain PS-218 Sp^R^ (at the distance of 3 cm) (Figure 2A) and allowed to swarm for 22-24 h at 37°C and elevated humidity (80% RH). We sampled 20 agar cores with a 1 ml trimmed pipette tip at the point where the common swarm met the opposing swarm (PS-216 Sp^R^ or PS-218 Sp^R^). The frequency of co-swarming strains was determined by plating on LB agar with selection for antibiotic resistance (MLS or Kn, respectively). We determined the competitive index (CI) of the focal strain PS-216 Δ*srfA* at the boundary with nonkin strain or at the merging point of kin strains. The competitive index (CI) was calculated by dividing Rf by Ri (Equation 1). The initial ratio between the two co-swarming strains (PS-216 Δ*srfA* and PS-216 Kn^R^) (Ri) was determined at the inoculum and the final ratio (Rf) at the swarm meeting point after swarming cycle was completed (Equation 1).

As a control experiment, we co-inoculated two swarming strains, the focal strain PS-216 Cm^R^ and the co-swarming strain PS-216 Kn^R^, and again staged the mixed swarm against the kin strain (PS-216 Sp^R^) or the nonkin strain (PS-218 Sp^R^) on a swarming medium followed by CFU determination as described above. As described above, we determined the ratio between the focal strain PS-216 Cm^R^ and the co-swarming PS-216 Kn^R^ in the inoculum and at the meeting area with a contact strain (kin PS-216 strain or nonkin PS-218 strain). The competitive index (CI) of a PS‑216 Cm^R^ strain was calculated (Equation 1). Contact strains’ cell abundances were disregarded. The experiment is described in greater detail in Supplementary 4.

Both experiments were performed in four independent experiments, each in three replicates.


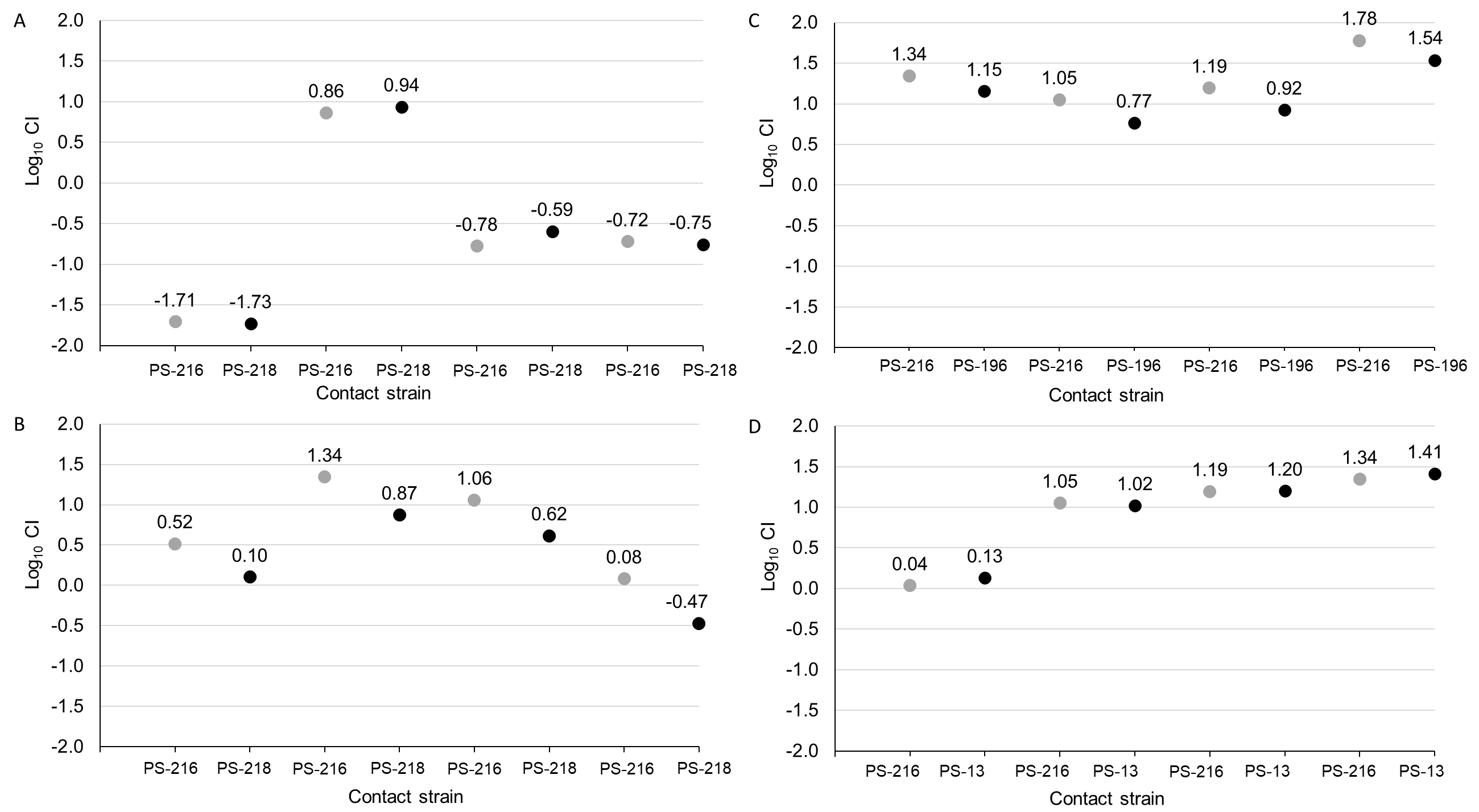


Supplementary 1 Figure S1: Competitive index (CI) of the control strain PS-216 Cm^R^ (A) or the surfactin non-producing mutant PS-216 ΔsrfA (B, C, and D), showing all four individual experiments in consecutive order, each performed in three replicates. (A) Competitive index (CI) of control strain PS-216 Cm^R^ swarming in a co-swarm with w.t. strain PS-216 Kn^R^ in contact with isogenic swarm PS-216 Sp^R^ (grey dots) or with nonkin strain PS-218 (black dots) (n=3). (B) Competitive index (CI) of non-swarming and surfactin non-producing mutant PS-216 ΔsrfA swarming in a co-swarm with w.t. strain PS-216 Kn^R^ when in contact with isogenic strain PS-216 Sp^R^ (grey dots) or with nonkin strain PS-218 Sp^R^ (black dots) (n=3). (C) Competitive index (CI) of the non-swarming and surfactin non-producing mutant PS-216 ΔsrfA swarming in a co-swarm with w.t. strain PS-216 Kn^R^ when in contact with isogenic strain PS-216 Sp^R^ (grey dots) or with nonkin strain PS-196 Sp^R^ (black dots) (n=3). (D) Competitive index (CI) of non-swarming and surfactin non-producing mutant PS-216 ΔsrfA swarming in a co-swarm with the wild-type strain PS-216 Kn^R^ when in contact with the isogenic strain PS-216 Sp^R^ (grey dots) or with the kin strain PS-13 Sp^R^ (black dots) (n=3).

The competitive index (CI) of the non-swarming and surfactin non-producing mutant PS-216 Δ*srfA* swarming in a common swarm with isogenic w.t. strain PS-216 Kn^R^ was higher in all four biological replicates when the common swarm was in contact with the isogenic strain PS-216, than when the common swarm was in contact with the nonkin swarm PS-218 Sp^R^ or the nonkin swarm PS-196 Sp^R^ (Supplementary 1 Figure S1B and Supplementary 1 Figure S1C, respectively). However, competitive index (CI) of the surfactin non-producing mutant PS-216 Δ*srfA* swarming in a co-swarm with the isogenic wild-type strain PS-216 Kn^R^ was similar in all four separate experiments, regardless of whether the common swarm was in contact with the isogenic strain PS-216 Sp^R^ or the kin strain PS-13 Sp^R^ (Supplementary Figure S1D). Similarly, the competitive index (CI) of the control strain PS-216 Cm^R^ swarming in a common swarm with the isogenic strain PS-216 Kn^R^ was similar regardless of the contact strain in all four experiments (Supplementary 1 Figure S1A).

**Supplementary 2: Number of evolved clones per evolved population screened for their swarming phenotype**

Supplementary 2 Table S1: Number of evolved clones per evolved population that were screened for their swarming phenotype after the 10^th^ or 20^th^ cycle of experimental evolution.

| **Contact** | **Population** | **All tested in 10th cycle** | **All tested in 20th cycle** |
| --- | --- | --- | --- |
| **PS-218** | A | 96 | 96 |
|  | B | 96 | 96 |
|  | C | 40 | 96 |
|  | D | 96 | 96 |
|  | E | not tested | 95 |
|  | F | not tested | 96 |
|  | G | not tested | 48 |
|  | H | not tested | 48 |
|  | I | not tested | 48 |
|  | J | not tested | 48 |
|  | K | not tested | 46 |
|  | L | not tested | 47 |
|  | M | not tested | 47 |
| **PS-13** | A | 96 | 96 |
|  | B | 82 | 96 |
|  | C | 96 | 96 |
|  | D | not tested | 96 |
|  | E | not tested | 95 |
|  | F | 96 | 96 |
|  | G | not tested | 47 |
|  | H | not tested | 48 |
|  | I | not tested | 48 |
|  | J | not tested | 48 |
|  | K | not tested | 48 |
|  | L | not tested | 47 |
|  | M | not tested | 48 |
|  | N | not tested | 48 |
| **PS-216** | A | 71 | 96 |
|  | B | 96 | 93 |
|  | C | 95 | 96 |
|  | D | 96 | 96 |
|  | E | not tested | 96 |
|  | F | not tested | 96 |
|  | G | not tested | 48 |
|  | H | not tested | 48 |
|  | I | not tested | 48 |
|  | J | not tested | 46 |
|  | K | not tested | 48 |
|  | L | not tested | 48 |

**Supplementary 3: Spread of PS-216 Δ*srfA* mutant strain in a common swarm**


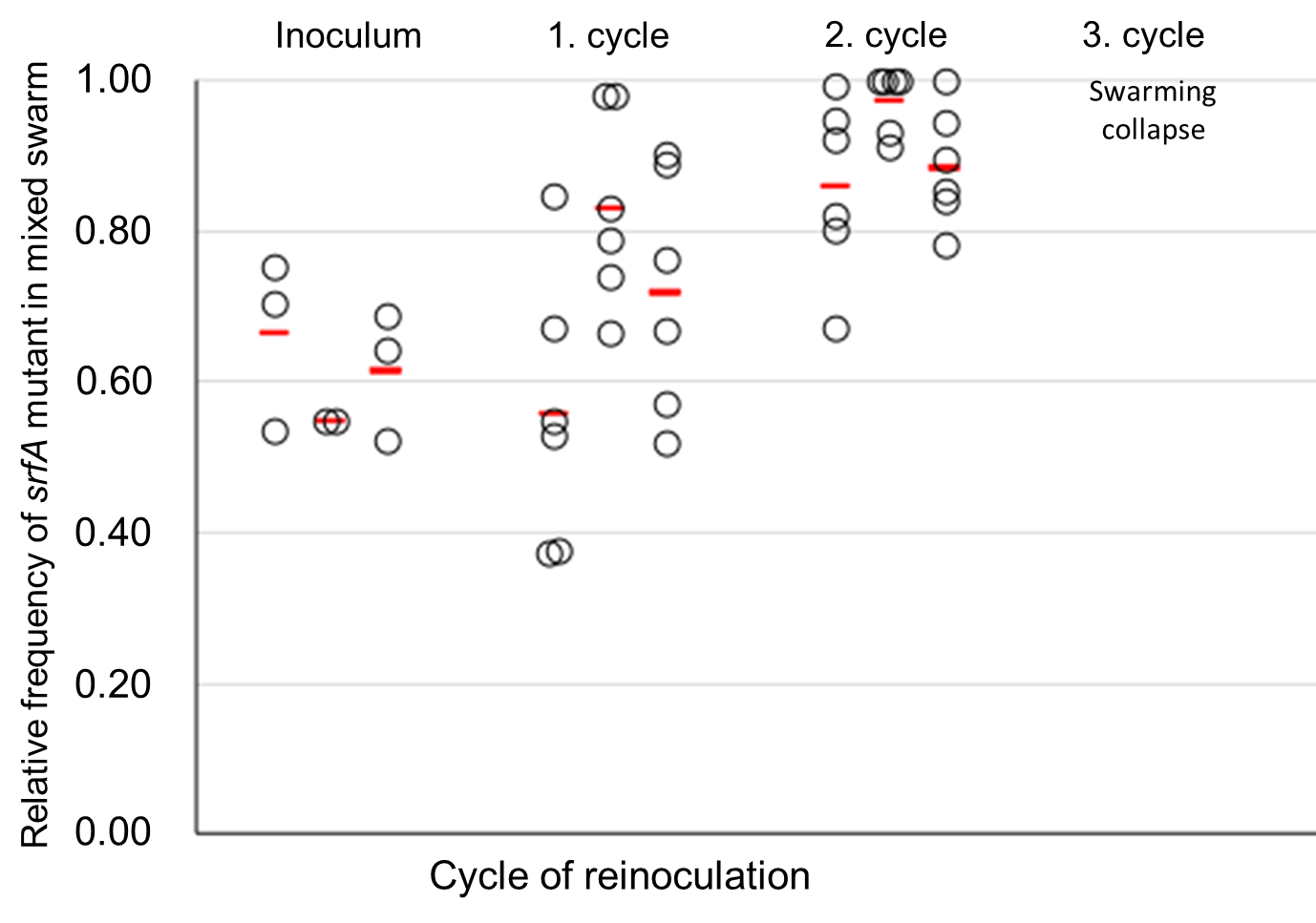


Supplementary 3 Figure S2: Relative frequency of PS-216 ΔsrfA mutant in inoculum and at the edge of the common swarm in each reinoculation cycle.

Mean relative frequency of PS-216 ΔsrfA mutant in inoculum or at the edge of the swarm in each reinoculation cycle for each separate experiment are presented (dash) (n=3) with each repeat presented as black circle (n=3 and n=6 for inoculum and each reinoculation cycle, respectively). No sampling was performed in 3^rd^ cycle due to swarming collapse.

**Supplementary 4: Relative competitive index of the swarming strain in a common swarm**

To test whether the observed effect of the nonkin interaction on the non-swarming mutant strain PS‑216 Δ*srfA* is indeed unique to the cheating strain, we performed a control experiment. The two swarming strains, the focal strain PS-216 Cm^R^ and the co-swarming strain PS-216 Kn^R^ were mixed at 1:1 ratio, staged at one side of the agar plate, and allowed to co-swarm against the kin strain (PS-216 Sp^R^) or the nonkin strain (PS-218 Sp^R^) on a swarming medium (Figure 2B) followed by CFU count of each strain according to antibiotic resistance (methods section of the main text). We determined the ratio between the focal strain PS‑216 Cm^R^ and the co-swarming PS-216 Kn^R^ in the inoculum and at the encounter area with a contact strain (kin PS-216 strain or nonkin PS-218 strain). Competitive index (CI) of a PS-216 Cm^R^ strain was calculated (Equation 1). The frequency of the contact strain cells were disregarded.

Due to the high variability between the CI for the focal strains (PS-216 Δ*srfA* or PS-216 Cm^R^) in four independent experiments (Supplementary 1 Figure S1) we determined the relative CI as the ratio between CI of the focal strain when common swarm was staged against kin or nonkin strain (CI_contact_) and CI of the focal strain when common swarm was staged against the isogenic contact strain (CI_isogenic contact_) (Equation 2). The ratios were consistent throughout independent experiments (Figure 2 and Supplementary 4 Figure S3).

Experiments were performed in four independent experiments, each with three replicates, and the relative competitive index was calculated for four independent experiments (n=4). (Supplementary 4 Figure S3).


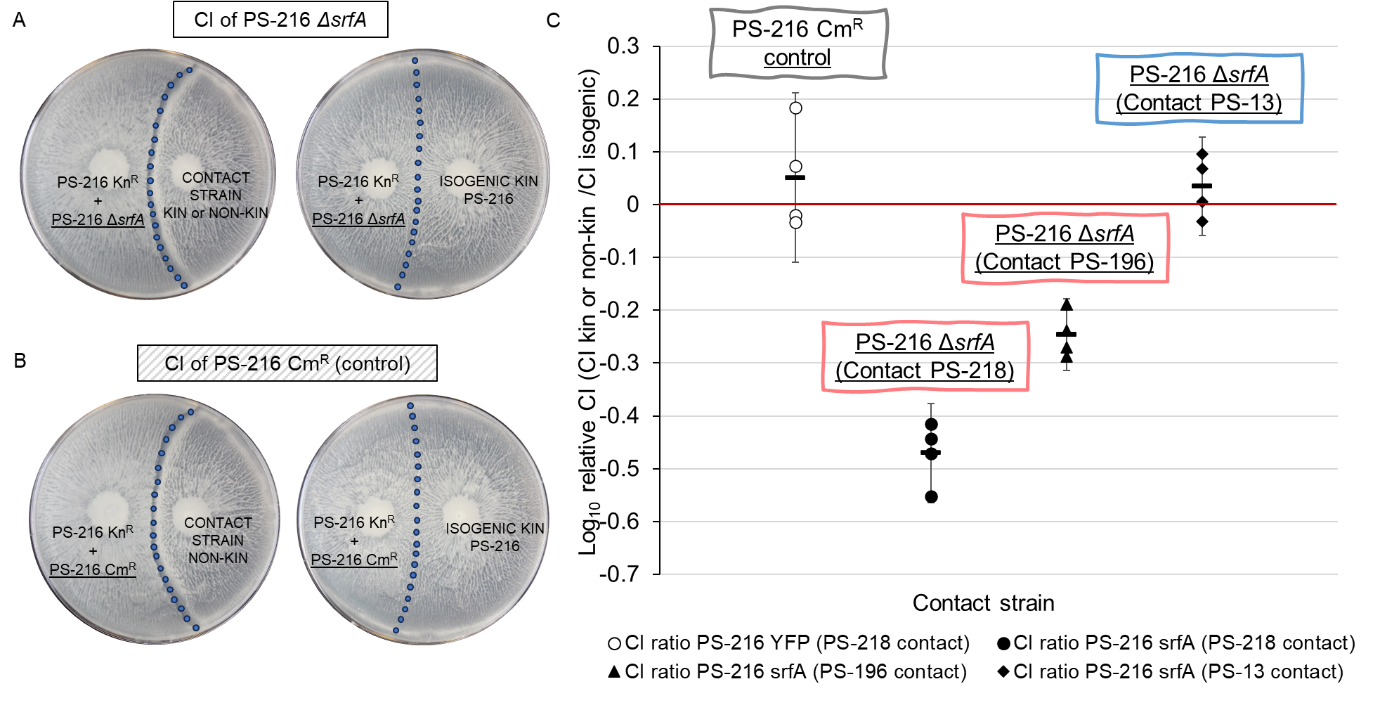


Supplementary 4 Figure S3: Relative competitive index of the surfactin mutant or wild-type strain PS-216 in a common swarm after contact with kin or nonkin swarm. A, B) Schematic representation of the experiment in which A) the surfactin non-producing mutant (PS-216 ΔsrfA) or B) swarming strain PS-216 Cm^R^ is mixed with the isogenic wild type strain (PS-216 Kn^R^) in 1:1 ratio and inoculated opposite to the isogenic strain (PS-216 Sp^R^) or contact strain (kin strain (PS-13 Sp^R^) or nonkin strain (PS-218 Sp^R^, PS-196 Sp^R^) Swarms were allowed to grow for 24 h and we sampled at the swarm encounter area. We determined CFU of each strain in the common swarm and calculated the competitive index (CI) of the focal strain. C) The relative CI represents the ratio between CI of focal strain (either PS-216 Cm^R^ or PS-216 ΔsrfA, underlined in schematic representation A and B) when the common swarm was staged against nonkin strain (CI_PS-218_ or CI_PS-196_) or kin contact strain (CI_PS-13_), and its CI when common swarm was staged against isogenic strain (CI_PS-216_) (Equation 2). The ratios were consistent throughout independent experiments. The relative CI was calculated for each of the four independent experiments, each performed in three replicates. The error bars represent the confidence interval (Ci) of four replicates(n=4).

**Supplementary 5: Competitive index of the *srfA* mutant at the contact area with isogenic or kin swarm relative to competitive index in the swarm area**

We determined the competitive index of the focal mutant PS-216 Δ*srfA* in a co-swarm with the wild-type PS-216 Kn^R^ at the contact with either the isogenic PS-216 Sp^R^ or the nonkin PS-218 Sp^R^ strain. In addition, the competitive index of the focal mutant PS-216 Δ*srfA* swarming with the wild-type PS‑216 Kn^R^ was determined at the swarm area approximately 1 cm from the inoculation point.

The overnight culture of PS-216 Δ*srfA* (MLS^R^) and PS-216 (Kn^R^) were mixed in 1:1 ratio and the relative frequency of each strain was determined by plating on LB agar with antibiotics according to resistance of each strain (MLS or Kn, respectively). CFUs were counted after overnight incubation at 37°C. During swarming assay, 2 µl of the mixed culture was inoculated opposite isogenic PS-216 Sp^R^ strain or nonkin strain PS-218 Sp^R^ (Figure 2A). Using a cut 1-ml pipette tip, 20 agar cores were collected at the point where the common swarm met the opposing swarm and approximately 1 cm from the inoculation site (in the swarm area) of the same common swarm. The samples were resuspended in 250 µl of saline solution (0.9% NaCl). Samples were vigorously mixed using a vortex mixer, and the relative frequency of co-swarming strains was again determined by plating on LB agar with selection for antibiotic resistance (MLS or Kn, respectively). We determined the competitive index (CI) of the focal strain PS-216 Δ*srfA* at both sampling sites. The competitive index (CI) was calculated by dividing the final ratio between the two co-swarming strains (PS-216 Δ*srfA* and PS-216 Kn^R^) in the swarm meeting area after completion of the swarming cycle or the ratio in the swarm area (*R_f_*) by the initial ratio between the two co-swarming strains (PS-216 Δ*srfA* and PS-216 Kn^R^) (*R_i_*) (Equation 1). The relative CI was determined by dividing CI of PS-216 Δ*srfA* at the isogenic or nonkin contact area by the CI within the swarm area of the same swarm (Supplementary 5 Equation S1 and Supplementary 5 Figure S4).

$${CI}_{relative} =\frac{\mathrm{CI}_{isogenic or non-kin contact point}}{\mathrm{CI}_{swarm area}}$$

Supplementary Equation S1

Experiments were performed in four independent experiments (n=4), each in three replicates. We calculated 95% confidence interval of the mean.


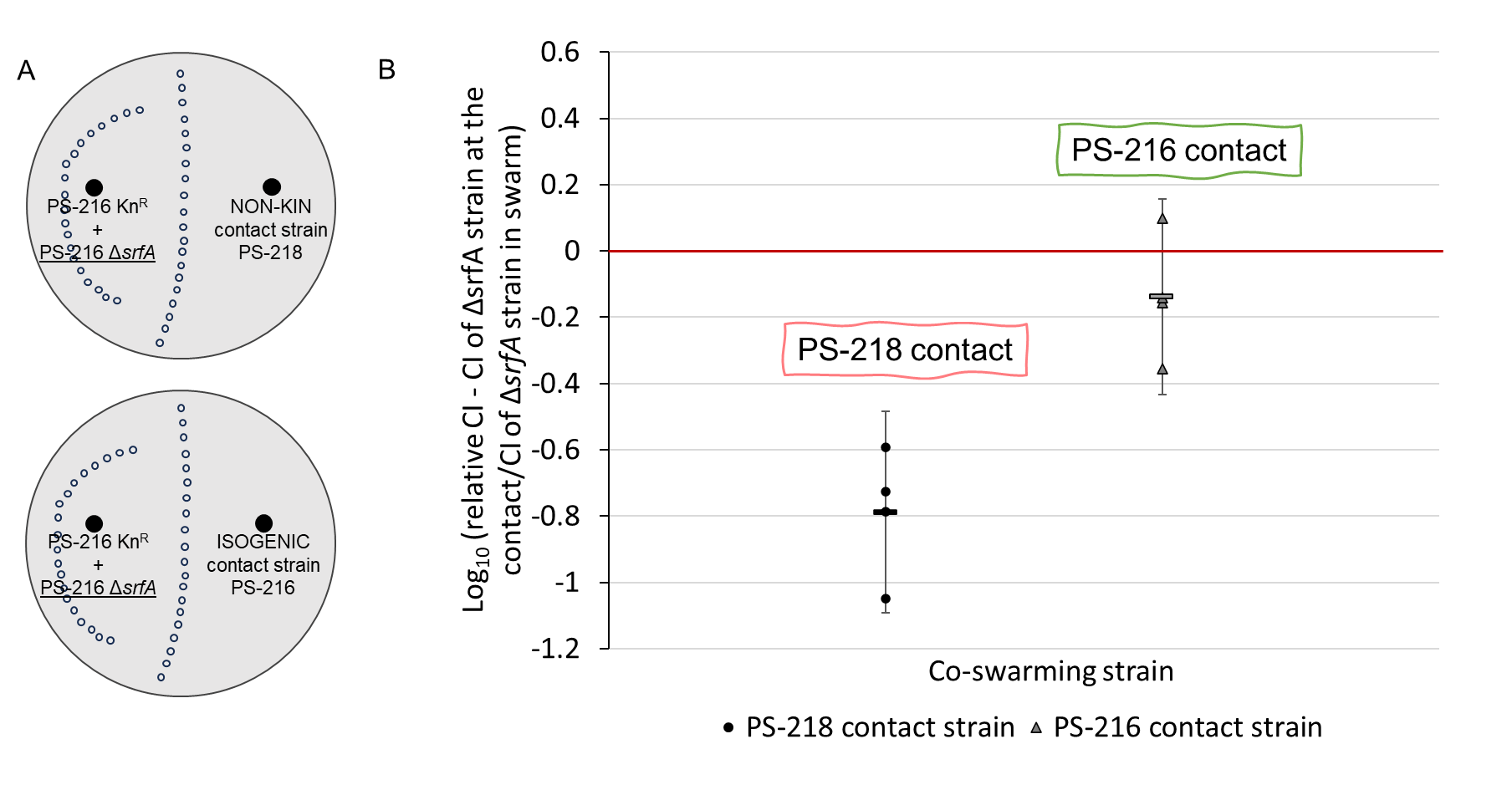


Supplementary 5 Figure S4: Relative competitive index of the surfactin mutant, calculated on the basis of CI of the surfactin mutant strain at the contact with an isogenic or nonkin strain in relation to its CI in the swarm area. A) Schematic representation of the experiment in which the surfactin non-producing mutant (PS-216 ΔsrfA) was mixed with the isogenic wild-type strain (PS-216 Kn^R^) in a 1:1 ratio and inoculated on one side of the agar opposite to the nonkin strain PS-218 Sp^R^ or the isogenic strain PS-216 Sp^R^ and allowed to swarm for 24 hr. We sampled at the swarm encounter area and 1 cm from inoculation point (in the swarm area) to determine the CFU of each strain in the common swarm and calculated the competitive index (CI). B) The relative CI represents the ratio between the CI of focal strain PS-216 ΔsrfA when the common swarm was staged against the nonkin strain (CI_PS-218_) or the isogenic contact strain (CI_PS-216_) and its CI 1 cm from the inoculation point of the same swarm (Equation 3). The relative CI was calculated for each of the four independent experiments, each performed in three replicates. The error bars represent the confidence interval (Ci) of four replicates (n=4).

**Supplementary 6: Growth rate (the number of generations)**

The microbial cultures were cultivated on LB agar plates and grown overnight at 37°C. One colony was transferred to liquid LB medium and shaken (200 rpm) for 16 h at 37°C. The next day, the culture was transferred to fresh liquid LB medium (1% inoculum) and shaken (200 rpm) at 37°C for 3 h to reach the exponential growth phase. 2 µl of the bacterial culture was inoculated into the center of the swarming agar and 3 µl of the culture was inoculated into 3 ml of the liquid LB medium. The number of cells in the inoculum was determined using the spread plate method (CFUs per milliliter). Inoculated swarming agar plates were incubated for 24 h at 37°C and 80% relative humidity (RH) and the inoculated liquid LB medium was shaken (200 rpm) for 24 h at 37°C. After incubation, 1 ml of a culture was sampled from the liquid LB, andthe cells on swarming agar were scraped from the agar surface and resuspended in 2 ml of saline solution (0.9% NaCl). The number of cells was determined using the spread plate method (CFUs per milliliter) in the inoculum (n_inoculum_) and after 24 h growth in liquid LB medium or on swarming agar (n_final_). The number of generations was calculated using Supplementary Equation S2.

$$No. of generations= \frac{\log\left( n_{final} \right)-\log(n_{inoculum})}{log2}$$

Supplementary Equation S2

The experiment was carried out in three separate experiments, each performed in six replicates. The mean of three seperate experiments and 95% confidence intervals of the means were calculated.


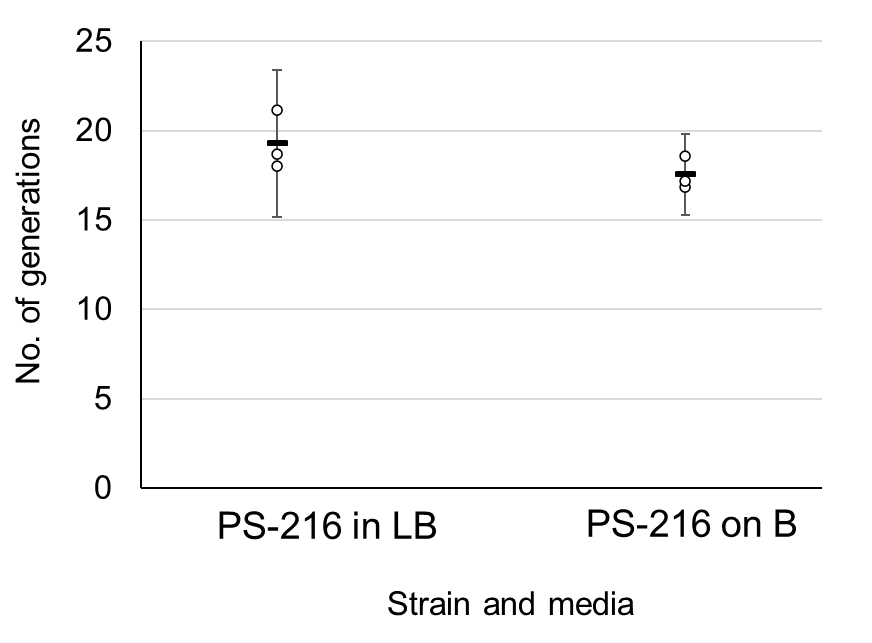


Supplementary 6 Figure S5: Number of generations of focal strain PS-216 that after 24 h of growth in liquid LB medium or after growth on swarming agar. Figure presents the mean with standard deviations of three independent experiments, each performed in six replicates. The error bars represent 95% confidence interval of mean values of the independent experiments (n=3).

**Supplementary 7: Mutation rate**

We determined the mutation rate of the focal strain PS-216 in liquid LB medium and on swarming agar. The frozen culture (-80°C) of the focal strain was scraped and transferred to saline solution (0.9% NaCl). The concentration of cells in the prepared cell suspension was determined using the spread plate method (CFUs per milliliter). The cell suspension was inoculated into the center of swarming agar plates (2 µl) and into the liquid LB medium (3 µl). Inoculated swarming agar plates were incubated at 37°C with 80% RH for 24 h, and the inoculated liquid medium was shaken (200 rpm) at 24 h at 37°C. After overnight incubation, 1 ml of liquid culture was sampled and the cells from the swarming agar were scraped and resuspended in 2 ml of saline solution (0.9% NaCl). The total number of cells in the liquid medium and on the surface of swarming agar after 24 h incubation was determined using the spread plate method (CFUs per milliliter). By selection for antibiotic resistance against rifampicin (Rif^R^) on LB agar media supplemented with rifampicin (5 µg/ml), we determined the total number of polymerase β subunit (*rpoB*) gene mutants that became resistant to rifampicin [1]. The experiment was performed in three independent experiments with six replicates. Mutation frequency was determined using the webtool for Luria-Delbrük experiment - webSalvador [2]. We used the Lea-Coulson Model with a plating efficiency of ε = 0.8 and estimated the final number of all cells (Nt) as the mean of the total number of cells after 24 h of six replicates, the initial number of mutations (Initial m) was set to zero and mutant counts were inserted. Using the integrated algorithm, the point estimate of the number of mutations (m) in 1 ml culture and the mutation rate (µ) were determined. The mean mutation rate of three separate experiments and 95% confidence intervals of the mean values were calculated.


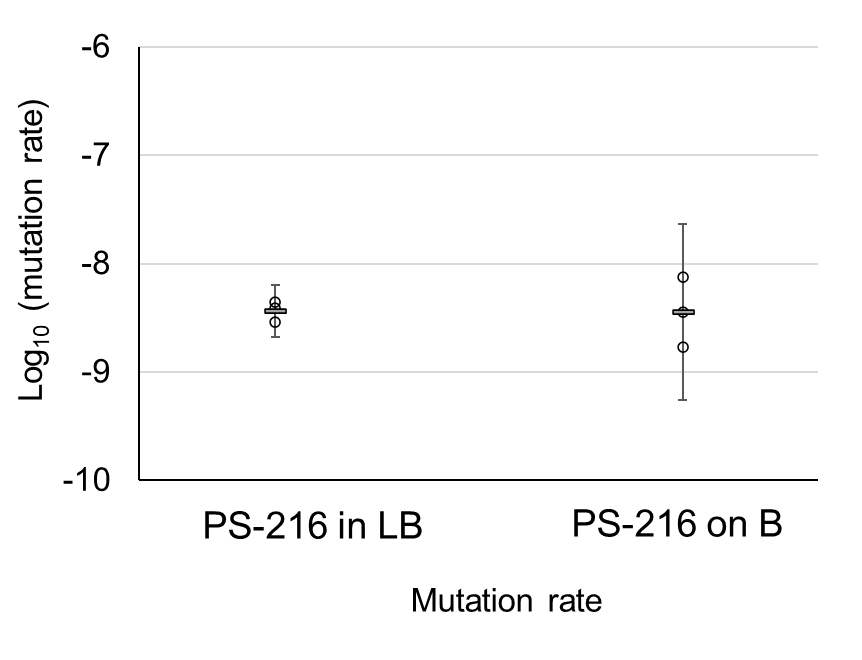


Supplementary Figure 7 S6: Mutation rate per cell per division determined by growing focal strain PS-216 in liquid LB medium or on semisolid swarming medium. Figure presents the mean with 95% confidence interval of mean for three independent experiments (n=3), each performed in six replicates.

**Supplementary 8: Swarming phenotype of evolved clones after 10^th^ cycle**


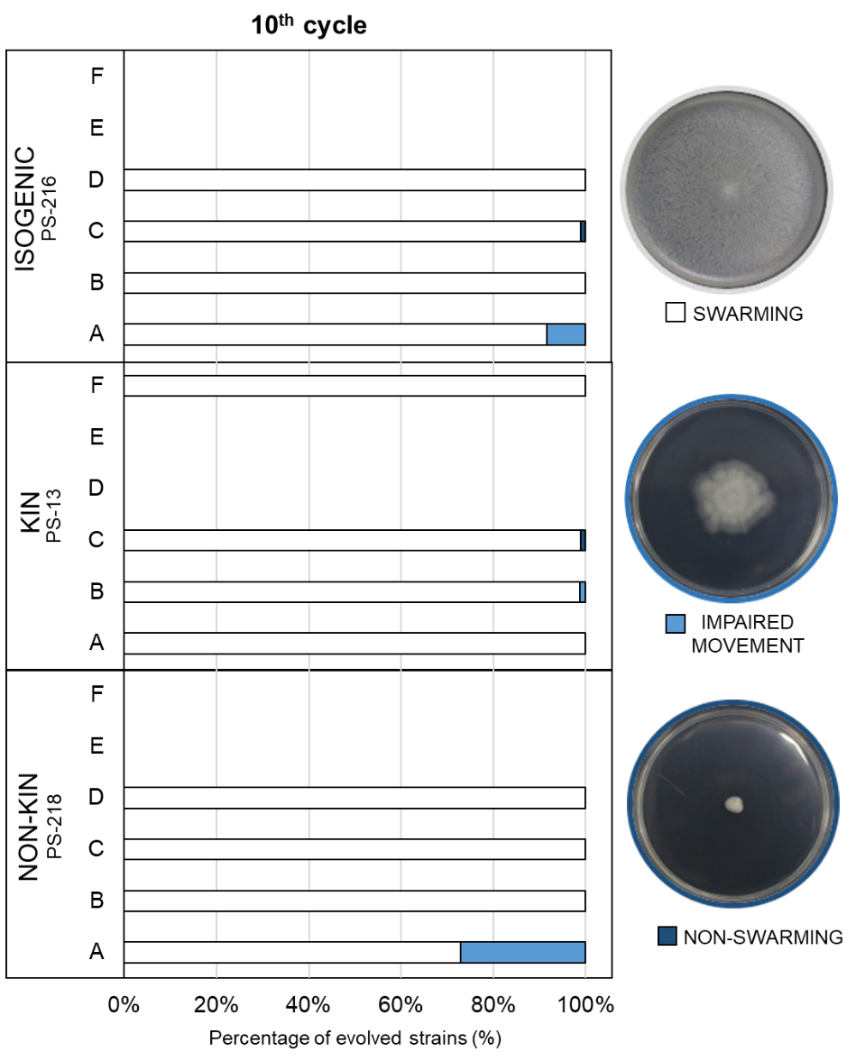


Supplementary 8 Figure S7: Swarming phenotypes of randomly selected evolved clones from 12 evolved populations in the 10th cycle (at least 40 evolved clones were screened per evolved population). Columns are coloured according to the relative frequency of each swarming phenotype for each evolved population. Images right of the graph are exemplifying the three swarming phenotypes.

**Supplementary 9: Competitive index of evolved clones and strain PS-216 Δ*srfA***

We revitalized the focal strains (evolved clones or the non-swarming mutant PS-216 Δ*srfA* (MLS^R^)) and the co-swarming strain PS-216 *sacA*::p43-*yfp* (Cm^R^) from frozen culture (-80°C) by streaking on LB (Lennox) agar plate and grown over night at 37°C. We inoculated grown cultures into liquid LB (Lennox) medium and incubated them for 16 hours at 37°C with shaking at 200 rpm to allow the cultures to grow. We mixed the culture of each focal strain with the culture of the co-swarming strain in a 1:1 ratio. We determined the concentration of each strain in the mixture using the CFU method. We inoculated the mixture (2 µl) into the center of the swarming agar plate. We incubated the plates at 37°C and increased humidity (80% RH) for 22-24 hours. After the swarms had formed, we sampled at the edge of the swarm (edge of the swarming agar) and again determined the concentration of the individual strains in the mixture using the CFU method. We determined the competitive index of the focal strain (evolved clone or non-swarming mutant PS-216 Δ*srfA*) by dividing the final ratio between focal strain and co-swarming strain in the inoculum (R _FINAL_) by the initial ratio between focal strain and co-swarming strain after completion of swarm growth by sampling at the swarm edge (R _INOCULUM_) (Equation 1). We performed the experiment in three replicates with the same inoculum.


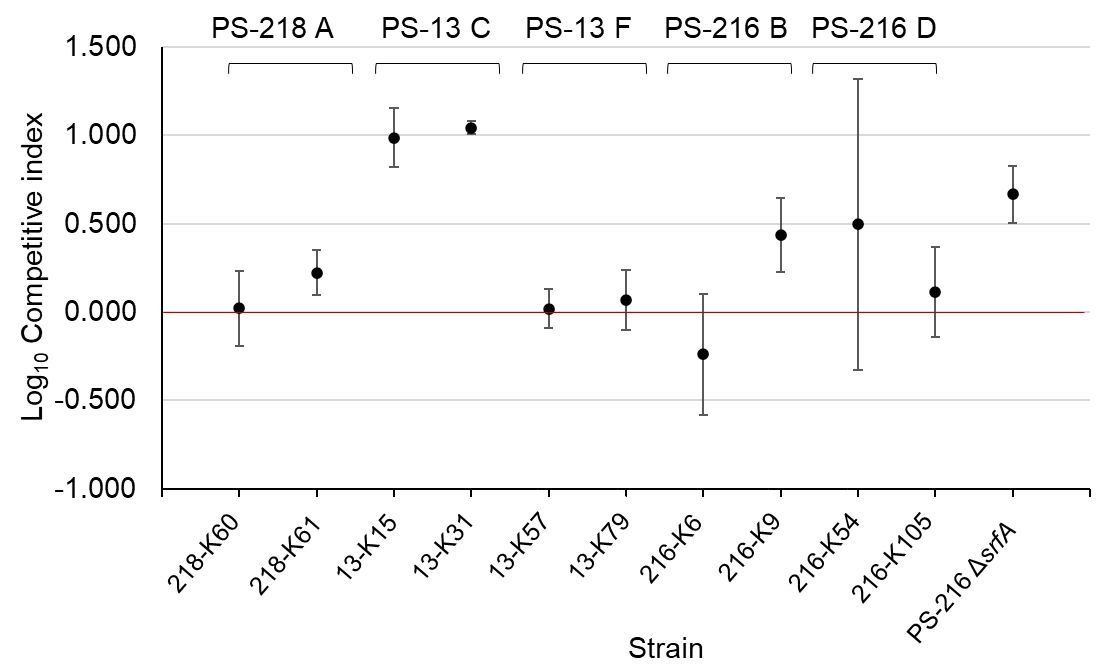


*Supplementary 9 Figure S8: Competitive index (log_10_ values) of evolved clones and the PS-216 ΔsrfA mutant strain which exhibited non-swarming phenotype. Results shown are the average competitive index of three repeats (n=3) with error bars representing the 95% confidence interval (Ci).*

**Supplementary 10:** **Results of independent sample T-test comparing surfactin concentrations produced by each evolved clone to the surfactin concentrations produced by parental strain PS‑216**

Supplementary 10 Table S2: Table of P values obtained by an independent sample T-test comparing the measured surfactin concentrations produced by each evolved clone with parental strain surfactin concentration measurements. P values were adjusted using the Bonferroni correction.

| Contact strain PS-216 | T-test *P* value | Contact strain PS-13 | T-test *P* value | Contact strain PS-218 | T-test *P* value |
| --- | --- | --- | --- | --- | --- |
| 216-K1 | 1.000 | 13-K60 | 1.000 | 218-K1 | 1.000 |
| 216-K4 | 1.000 | 13-K61 | 1.000 | 218-K3 | 1.000 |
| 216-K7 | 1.000 | 13-K80 | 1.000 | 218-K7 | 1.000 |
| 216-K10 | 1.000 | 13-K83 | 1.000 | 218-K10 | 1.000 |
| 216-K12 | 1.000 | 13-K85 | 1.000 | 218-K12 | 1.000 |
| 216-K15 | 0.020 | 13-K86 | 1.000 | 218-K16 | 1.000 |
| 216-K59 | 1.000 | 13-K88 | 1.000 | 218-K20 | 1.000 |
| 216-K106 | 1.000 | 13-K87 | 0.040 | 218-K24 | 1.000 |
| 216-K107 | 1.000 | 13-K84 | 0.001 | 218-K27 | 1.000 |
| 216-K108 | 1.000 | 13-K82 | <0.001 | 218-K30 | 1.000 |
| 216-K109 | 1.000 | 13-K1 | <0.001 | 218-K32 | 0.040 |
| 216-K110 | 1.000 | 13-K8 | <0.001 | 218-K56 | 1.000 |
| 216-K111 | 1.000 | 13-K27 | <0.001 | 218-K57 | 1.000 |
| 216-K112 | 1.000 | 13-K15 | <0.001 | 218-K58 | 1.000 |
| 216-K113 | 1.000 | 13-K20 | <0.001 | 218-K60 | 1.000 |
| 216-K3 | <0.001 | 13-K31 | <0.001 | 218-K61 | 1.000 |
| 216-K6 | <0.001 | 13-K41 | <0.001 | 218-K62 | 1.000 |
| 216-K9 | <0.001 | 13-K57 | <0.001 | 218-K63 | 1.000 |
| 216-K16 | <0.001 | 13-K66 | <0.001 | 218-K59 | 0.200 |
| 216-K25 | <0.001 | 13-K79 | <0.001 | 218-K2 | <0.001 |
| 216-K36 | <0.001 | 13-K81 | <0.001 |  |  |
| 216-K42 | <0.001 |  |  |  |  |
| 216-K54 | <0.001 |  |  |  |  |
| 216-K89 | <0.001 |  |  |  |  |
| 216-K105 | <0.001 |  |  |  |  |

**Supplementary 11: Average surfactant concentration of the evolved clones and their swarming phenotype**

Supplementary 11 Table S3: Table of average surfactant concentrations determined according to the Drop collapse assay for each evolved clone. The clones’ contact strain during experimental evolution is indicated on the left and a more detailed description of the swarming phenotype in the fifth column.

| Contact strain | Population | Clone | Average surfactant concentration (µg/ml) | Swarming phenotype |
| --- | --- | --- | --- | --- |
| PS-216 | A | 216-K1 | 101.2 | Non-dendritic swarming |
|  | A | 216-K4 | 114.7 | Non-dendritic swarming |
|  | C | 216-K7 | 134.6 | Non-dendritic swarming |
|  | A | 216-K10 | 140.6 | Swarming |
|  | E | 216-K12 | 102.6 | Swarming |
|  | E | 216-K59 | 96.6 | Swarming |
|  | F | 216-K106 | 96.9 | Swarming |
|  | E | 216-K107 | 104.0 | Swarming |
|  | E | 216-K108 | 121.6 | Swarming |
|  | F | 216-K109 | 117.7 | Swarming |
|  | E | 216-K110 | 101.3 | Swarming |
|  | E | 216-K111 | 112.3 | Swarming |
|  | E | 216-K112 | 126.1 | Swarming |
|  | E | 216-K113 | 115.8 | Swarming |
|  | B | 216-K3 | 4.3 | Non-dendritic swarming |
|  | B | 216-K6 | 8.7 | Non-dendritic swarming |
|  | B | 216-K9 | 3.3 | Non-dendritic swarming |
|  | E | 216-K15 | 183.5 | Swarming |
|  | D | 216-K16 | 2.2 | Non-swarming |
|  | E | 216-K25 | 4.1 | Non-swarming |
|  | D | 216-K36 | 3.3 | Non-swarming |
|  | D | 216-K42 | 3.5 | Non-swarming |
|  | D | 216-K54 | 3.4 | Non-swarming |
|  | F | 216-K89 | 4.7 | Non-swarming |
|  | D | 216-K105 | 2.9 | Non-swarming |
| PS-13 | D | 13-K60 | 88.4 | Swarming |
|  | D | 13-K61 | 90.7 | Swarming |
|  | D | 13-K80 | 129.3 | Swarming |
|  | D | 13-K83 | 117.8 | Swarming |
|  | D | 13-K85 | 89.1 | Swarming |
|  | D | 13-K86 | 100.3 | Swarming |
|  | D | 13-K88 | 98.5 | Swarming |
|  | D | 13-K87 | 56.7 | Swarming |
|  | F | 13-K84 | 25.9 | Swarming |
|  | F | 13-K79 | 3.0 | Non-swarming |
|  | E | 13-K82 | 4.0 | Swarming |
|  | C | 13-K1 | 2.3 | Impaired movement |
|  | C | 13-K8 | 2.8 | Impaired movement |
|  | C | 13-K27 | 2.0 | Non-swarming |
|  | C | 13-K15 | 3.1 | Non-swarming |
|  | C | 13-K20 | 2.3 | Impaired movement |
|  | C | 13-K31 | 1.8 | Impaired movement |
|  | F | 13-K41 | 2,8 | Non-swarming |
|  | F | 13-K57 | 3.4 | Non-swarming |
|  | F | 13-K66 | 2.4 | Non-swarming |
|  | F | 13-K81 | 4.0 | Non-swarming |
| PS-218 | A | 218-K1 | 126.8 | Swarming |
|  | D | 218-K3 | 110.4 | Non-dendritic swarming |
|  | D | 218-K7 | 123.8 | Non-dendritic swarming |
|  | D | 218-K10 | 112.8 | Non-dendritic swarming |
|  | D | 218-K12 | 119.7 | Swarming |
|  | F | 218-K16 | 100.2 | Non-dendritic swarming |
|  | F | 218-K20 | 95.4 | Non-dendritic swarming |
|  | F | 218-K24 | 104.8 | Non-dendritic swarming |
|  | F | 218-K27 | 95.4 | Non-dendritic swarming |
|  | F | 218-K30 | 142.7 | Non-dendritic swarming |
|  | F | 218-K32 | 195.1 | Swarming |
|  | D | 218-K56 | 114.3 | Swarming |
|  | E | 218-K57 | 81.3 | Swarming |
|  | D | 218-K58 | 119.1 | Swarming |
|  | D | 218-K60 | 129.0 | Swarming |
|  | D | 218-K61 | 117.3 | Swarming |
|  | D | 218-K62 | 86.1 | Swarming |
|  | D | 218-K63 | 93.4 | Swarming |
|  | E | 218-K59 | 61.7 | Swarming |
|  | A | 218-K2 | 11.7 | Impaired movement |

**Supplementary 12: Surface tension measurements of evolved populations’ in spent medium**

Frozen (-80°C) evolved populations were inoculated into liquid LB with added spectinomycin and kanamycin and grown for 16 h at 37°C with shaking (200 rpm). The grown cultures were centrifuged for 5 min at 10 000 g and filtered (0.2 µm pores) to remove all cells. The surface tension of the spent media was determined using the Krüss Drop Shape Analyzer DSA25 (Hamburg, Germany), which employs the pendant drop technique to measure surface tension. A pendant drop of each sample was formed from a flat cannula, the silhouette of an axisymmetric fluid droplet was captured after 60 s of incubation when equilibrium was reached, and the Young-Laplace equation was used to calculate the surface tension of the samples. The surface tension of MQ was also determined as described above. Surface tension of three separate droplets was measured for each spent media sample, and the confidence interval (Ci) was calculated for three measurements.


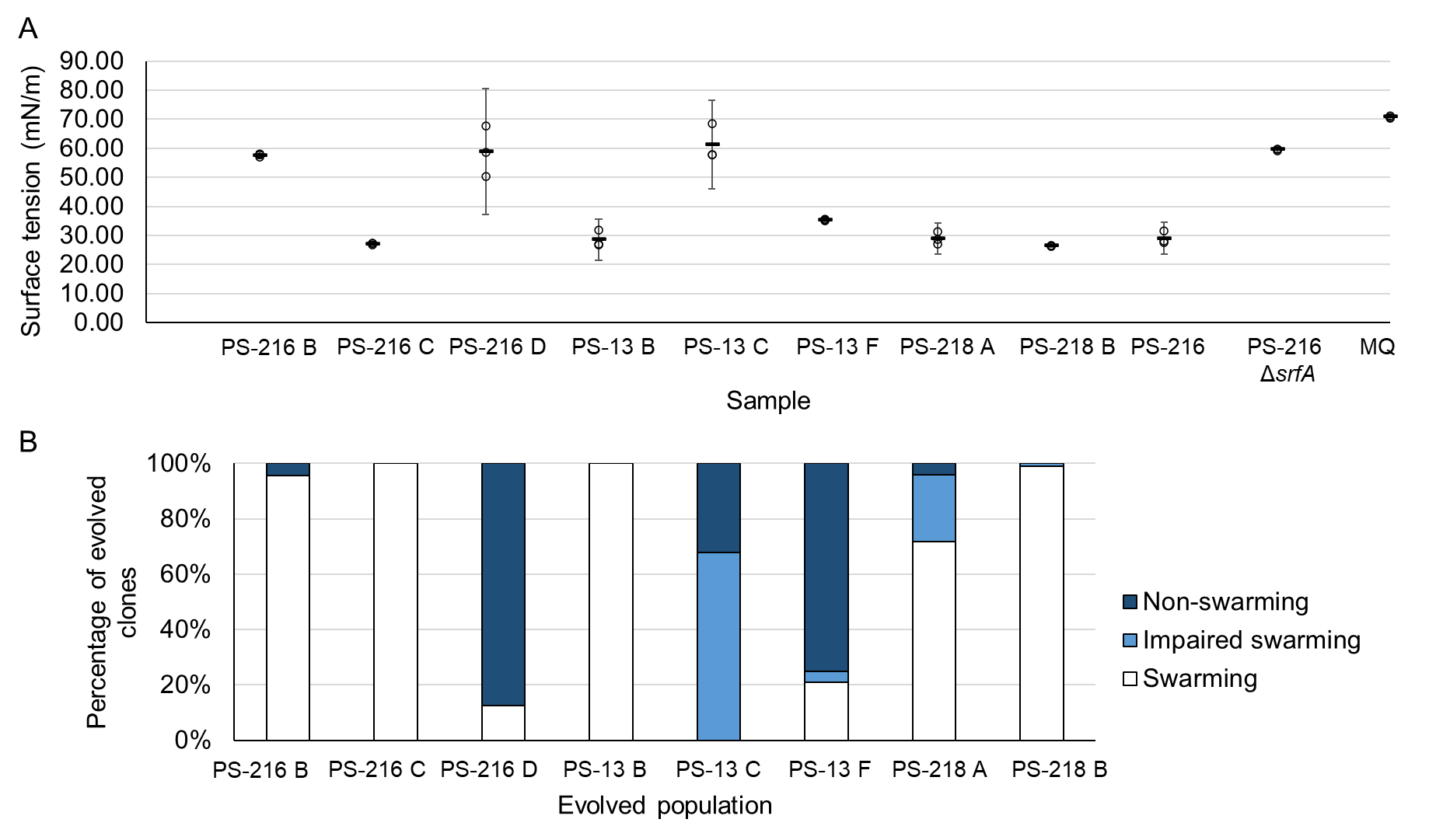


Supplementary 12 Figure S9: Surface tension measurements of the spent medium of the evolved populations of the wild-type strain PS-216 Sp^R^ Kn^R^ and the non-swarming mutant strain PS-216 ΔsrfA after 16 hours of growth in LB medium and the evolved populations’ structure according to the swarming phenotype of the evolved clones. A) Measured surface tension of the spent medium of the evolved populations and the non-swarming mutant strain PS-216 ΔsrfA after 16 h of growth in LB medium (n=3) (circles). The average surface tension is shown as black lines, with the error bars representing the confidence intervals. Additionally, measurements were also carried out for MQ water as a control (n=3). The measured low surface tension indicates that surfactants such as surfactin are present in the spent medium, whereas a high surface tension (as MQ water) suggest that none or little surfactants are present. B) Evolved populations’ structure according to the swarming phenotype of the evolved clones for each population for which surface tension measurements of the spent medium were performed.

Supplementary references:

1. Nicholson W. L. & Maughan H. The spectrum of spontaneous rifampin resistance mutations in the *rpoB* gene of *Bacillus subtilis* 168 spores differs from that of vegetative cells and resembles that of *Mycobacterium tuberculosis*. *J Bacteriol* 2002; **184**: 4936–4940. <https://doi.org/10.1128/jb.184.17.4936-4940.2002>

2. Zheng Q. webSalvador: a web tool for the Luria-Delbrük experiment. *Microbiol Resour Announc* 2021; **10**: e00314-21. <https://doi.org/10.1128/mra.00314-21>
